# Increasing cocoa butter-like lipid production of *Saccharomyces cerevisiae* by expression of selected cocoa genes

**DOI:** 10.1186/s13568-017-0333-1

**Published:** 2017-02-06

**Authors:** Yongjun Wei, Michael Gossing, David Bergenholm, Verena Siewers, Jens Nielsen

**Affiliations:** 1 Department of Biology and Biological Engineering, Chalmers University of Technology, SE-41296 Gothenburg, Sweden; 2 Novo Nordisk Foundation Center for Biosustainability, Chalmers University of Technology, SE-41296 Gothenburg, Sweden; 3 Novo Nordisk Foundation Center for Biosustainability, Technical University of Denmark, 2800 Kongens Lyngby, Denmark

**Keywords:** Phylogenetic analysis, TAG biosynthetic genes, Cocoa butter-like lipids, Metabolic engineering, Synthetic biology, Cell factories

## Abstract

Cocoa butter (CB) extracted from cocoa beans mainly consists of three different kinds of triacylglycerols (TAGs), 1,3-dipalmitoyl-2-oleoyl-glycerol (POP, C16:0–C18:1–C16:0), 1-palmitoyl-3-stearoyl-2-oleoyl-glycerol (POS, C16:0–C18:1–C18:0) and 1,3-distearoyl-2-oleoyl-glycerol (SOS, C18:0–C18:1–C18:0), but CB supply is limited. Therefore, CB-like lipids (CBL, which are composed of POP, POS and SOS) are in great demand. *Saccharomyces cerevisiae* produces TAGs as storage lipids, which are also mainly composed of C16 and C18 fatty acids. However, POP, POS and SOS are not among the major TAG forms in yeast. TAG synthesis is mainly catalyzed by three enzymes: glycerol-3-phosphate acyltransferase (GPAT), lysophospholipid acyltransferase (LPAT) and diacylglycerol acyltransferase (DGAT). In order to produce CBL in *S. cerevisiae*, we selected six cocoa genes encoding GPAT, LPAT and DGAT potentially responsible for CB biosynthesis from the cocoa genome using a phylogenetic analysis approach. By expressing the selected cocoa genes in *S. cerevisiae*, we successfully increased total fatty acid production, TAG production and CBL production in some *S. cerevisiae* strains. The relative CBL content in three yeast strains harboring cocoa genes increased 190, 230 and 196% over the control strain, respectively; especially, the potential SOS content of the three yeast strains increased 254, 476 and 354% over the control strain. Moreover, one of the three yeast strains had a 2.25-fold increased TAG content and 6.7-fold higher level of CBL compared with the control strain. In summary, CBL production by *S. cerevisiae* were increased through expressing selected cocoa genes potentially involved in CB biosynthesis.

**Electronic supplementary material:**

The online version of this article (doi:10.1186/s13568-017-0333-1) contains supplementary material, which is available to authorized users.

## Introduction

Cocoa butter (CB) extracted from cocoa seeds (*Theobroma cacao*) mainly contains three different kinds of triacylglycerols (TAGs)—1,3-dipalmitoyl-2-oleoyl-glycerol (POP, C16:0–C18:1–C16:0), 1-palmitoyl-3-stearoyl-2-oleoyl-glycerol (POS, C16:0–C18:1–C18:0) and 1,3-distearoyl-2-oleoyl-glycerol (SOS, C18:0–C18:1–C18:0), which are composed of C16 and C18 fatty acids (Jahurul et al. 2013). CB is mainly used for chocolate production and there is a world-wide increasing demand for chocolate (Clough et al. 2009). However, the CB supply is limited because cocoa trees only grow in a limited geographical zone and diseases affect cocoa trees nearly every year (Clough et al. 2009). Therefore, finding new sustainable sources for CB ingredients, such as CB-like lipids (CBL, which are composed of POP, POS and SOS), is of interest. TAGs are used as energy and carbon storage in yeasts (Sorger and Daum 2003), such as the model *Saccharomyces cerevisiae* and the main components of its TAGs are usually C16 and C18 fatty acids (Koch et al. 2014; Sorger and Daum 2003), suggesting that *S. cerevisiae* has the potential to be used for CBL production.

**Fig. 1:**
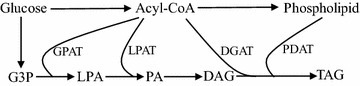
Three Enzymes, GPAT, LPAT, DGAT, determine the TAG structure in the TAG biosynthetic pathway. *G3P* glycerol-3-phosphate, *LPA* lysophosphatidic acid, *PA* phosphatidic acid, *DAG* diacylglycerol, *TAG* triacylglycerol, *GPAT* glycerol-3-phosphate acyltransferase, *LPAT* lysophosphatidic acid acyltransferase, *DGAT* acyl-CoA: diacylglycerol acyltransferase, *PDAT* phospholipid: diacylglycerol acyltransferase

Triacylglycerol is an ester composed of one glycerol and three fatty acids, and its synthesis is mainly catalyzed by three different kinds of enzymes: glycerol-3-phosphate acyltransferase (GPAT), lysophospholipid acyltransferase (LPAT) and diacylglycerol acyltransferase (DGAT), which can add acyl-coenzyme As (acyl-CoAs) to the *sn*-1, *sn*-2 and *sn*-3 position of glycerol, respectively (Chapman and Ohlrogge 2012). *S. cerevisiae* contains two GPATs (Gpt2p and Sct1p), two LPATs (Slc1p and Slc4p) and one DGAT (Dga1p). Additionally, another phospholipid:diacylglycerol acyltransferase, PDAT (Lro1p), can also synthesize TAG using diacylglycerol (DAG) and phospholipid as substrates (Fig. 1) (Coleman and Lee 2004; de Kroon et al. 2013; Ratledge 2002; Zheng and Zou 2001). Previous studies showed that double deletions of either the two GPAT genes or the two LPAT genes of *S. cerevisiae* were lethal, indicating these genes are essential in yeast (Benghezal et al. 2007; Zheng and Zou 2001). Though C16:0, C16:1, C18:0 and C18:1 are the four main fatty acids in the total fatty acid composition of *S. cerevisiae* (Khoomrung et al. 2012), only small amounts of CBL (POP, POS, SOS) have been identified among the TAGs in wild-type *S. cerevisiae* cells (Ejsing et al. 2009), suggesting that its GPAT, LPAT and DGAT enzymes might not be optimal for CBL production.

As natural CB or its equivalents are mainly derived from plant fruits or seeds, global analyses of some plant GPAT, LPAT and DGAT genes, could reveal potential CB biosynthetic genes (Jahurul et al. 2013). However, different from yeast, plants usually contain many GPAT, LPAT and DGAT genes. For example, there are ten GPAT genes, nine LPAT genes and four DGAT genes in the genome of the model plant *Arabidopsis thaliana* (Chen et al. 2011; Kim et al. 2005; Turchetto-Zolet et al. 2011). The GPAT, LPAT and DGAT genes of *A. thaliana* are diverse (Chen et al. 2011; Kim et al. 2005) and some of them are functionally redundant, such as *GPAT4* and *GPAT8*. Only a double knockout of both *GPAT4* and *GPAT8* can strongly reduce cutin biosynthesis in *A. thaliana* (Li et al. 2007). It is therefore difficult to select GPAT, LPAT and DGAT genes specific for CB biosynthesis directly from plants. As genome information of *T*. *cacao* is available (Argout et al. 2011; Motamayor et al. 2013), recovering GPAT, LPAT and DGAT genes of *T. cacao*, which are potentially responsible for CB biosynthesis, and expressing them in *S. cerevisiae* might provide functional information and at the same time enable improved CBL production in this yeast. Two DGAT genes of *T*. *cacao* have been characterized and described by Zhang (Zhang 2012), and their expression in a yeast strain deficient in TAG synthesis led to accumulation of TAGs different from the wild-type strain.

Here we retrieved six potential CB biosynthetic genes (two GPAT, two LPAT and two DGAT genes) in *T. cacao* using a phylogenetic analysis approach. In order to verify the function of these cocoa genes and to understand their effects on lipid production of *S. cerevisiae*, we expressed them individually or combined in *S. cerevisiae* and compared the total fatty acid content in the engineered yeasts. Based on the total fatty acid results, we selected three strains harboring cocoa genes for further analysis of the total lipid composition and the TAG profile, and compared these with the similar measurements for the control strain. This analysis provided new functional insight into CB biosynthetic enzymes and advanced CBL production using *S. cerevisiae* as a cell factory.

## Materials and methods

### Strains and plasmids

The *Escherichia coli* strain DH5α was used for all the cloning work in this study, and the *E. coli* transformants were selected on LB medium containing 100 µg ml^−1^ ampicillin. The *S. cerevisiae* strain used was CEN.PK 113-11C (*MAT*a *MAL2*-*8c SUC2 ura3*-*52 his3*-*Δ1*), which was kindly provided by Kötter Entian and Kötter (2007). The strains harboring cocoa genes were constructed based on *S. cerevisiae* CEN.PK 113-11C and all the yeast strains constructed in this study are listed in Table 1. Yeast strains harboring cocoa genes were selected on synthetic complete (SC) dropout media (Formedium Ltd) (Li et al. 2015). The minimal medium, containing 7.5 g l^−1^ (NH_4_)_2_SO_4_, 14.4 g l^−1^ KH_2_PO_4_, 0.5 g l^−1^ MgSO_4_·7H_2_O, 20 g l^−1^ glucose, trace metal solution and vitamin solution (Verduyn et al. 1992), supplemented with 100 mg l^−1^ histidine, was used for 20 ml shake flask batch cultivation. The nitrogen-limited medium (named NLM medium in the text) was used for 1 l shake flask batch cultivations (Yang et al. 2014).

**Table 1:** List of strains derived from *S. cerevisiae* CEN.PK 113-11C and used in this study

Name	Expression plasmids	Properties
YJ0	pBS01A	Empty vector
YJ-G01	pYJ-G01	*TcGPAT1* expression
YJ-G02	pYJ-G02	*TcGPAT2* expression
YJ-L01	pYJ-L01	*TcLPAT1* expression
YJ-L02	pYJ-L02	*TcLPAT2* expression
YJ-D01	pYJ-D01	*TcDGAT1* expression
YJ-D02	pYJ-D02	*TcDGAT2* expression
YJ-111	pYJ-111	*TcGPAT1*, *TcLPAT1* and *TcDGAT1* gene combination expression
YJ-112	pYJ-112	*TcGPAT1*, *TcLPAT1* and *TcDGAT2* gene combination expression
YJ-121	pYJ-121	*TcGPAT1*, *TcLPAT2* and *TcDGAT1* gene combination expression
YJ-122	pYJ-122	*TcGPAT1*, *TcLPAT2* and *TcDGAT2* gene combination expression
YJ-211	pYJ-211	*TcGPAT2*, *TcLPAT1* and *TcDGAT1* gene combination expression
YJ-212	pYJ-212	*TcGPAT2*, *TcLPAT1* and *TcDGAT2* gene combination expression
YJ-221	pYJ-221	*TcGPAT2*, *TcLPAT2* and *TcDGAT1* gene combination expression
YJ-222	pYJ-222	*TcGPAT2*, *TcLPAT2* and *TcDGAT2* gene combination expression

### Phylogenetic analysis of cocoa GPAT, LPAT and DGAT genes

The GPAT, LPAT and DGAT gene sequences of *T*. *cacao* annotated by CGD (Cacao Genome Database, http://www.cacaogenomedb.org/) and KEGG databases were downloaded from the Genbank database (Kanehisa et al. 2016). Reference GPAT, LPAT and DGAT sequences of *A*. *thaliana*, *Homo sapiens* and *S. cerevisiae* were directly downloaded from the KEGG database (Kanehisa et al. 2016). Multiple alignments of amino acid sequences of GPATs, LPATs or DGATs were carried out using the MAFFT online version (Katoh and Standley 2013). The alignment results were used to create phylogenetic trees using the MEGA 6.06 software, and the used method was the Neighbor-Joining method with Poisson correction (Tamura et al. 2013). The bootstrap confidence values were based on 1000 replicates. The pair wise-deletion option was used to treat gaps in the alignment of GPAT, LPAT or DGAT sequences. Two cocoa GPAT, two cocoa LPAT and two cocoa DGAT genes which were similar to characterized TAG biosynthetic genes were selected as potential CB biosynthetic genes and used for expression in *S. cerevisiae*.

### Synthesis of cocoa genes and expression plasmid construction

Six cocoa genes encoding GPAT, LPAT or DGAT were synthesized codon-optimized for expression in *S. cerevisiae* (GeneArt Gene Synthesis, Thermo Fisher Scientific). The six synthesized cocoa gene sequences were deposited at the GenBank database under the accession number of KX982578–KX982583.

**Fig. 2:**
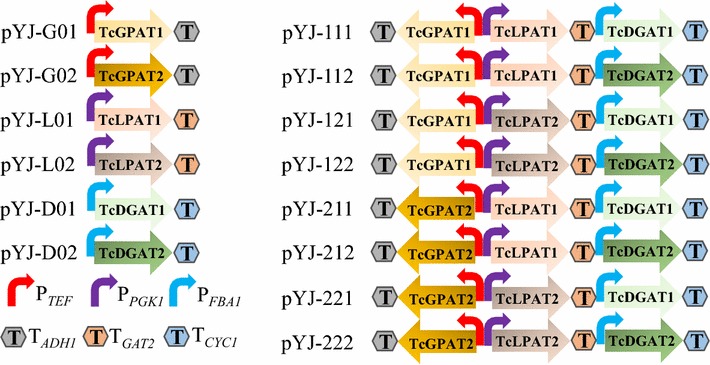
Schematic organization of cocoa gene expression cassettes in each of the expression plasmids

The primers used to amplify cocoa genes, promoters and terminators are listed in Additional file 1: Table S1. The cocoa genes were amplified from the synthesized genes; Promoter P_*TEF1*_ of *Ashbya gossypii* was amplified from template pUG60 (Goldstein et al. 1999); Promoters P_*PGK1*_ and P_*FBA1*_, and terminators T_*ADH1*_, T_*GAT2*_ and T_*CYC1*_ were amplified from genomic DNA of *S. cerevisiae* CEN.PK 113-11C. The backbone fragment of plasmid pBS01A was amplified from the expression vector of pBS01A (derived from pSP-GM1, see the Additional file 1) (Chen et al. 2012). Promoters, cocoa genes and terminators were fused into cocoa gene expression cassettes using overlap extension PCR (Zhou et al. 2012). The gene expression cassettes were verified by PCR and the structure of all the cassettes is described in Fig. 2. The verified gene expressing cassettes were ligated into the amplified backbone fragment of plasmid pBS01A using the Gibson assembly method (NEB) to construct cocoa gene expression plasmids, which were verified with PCR and Sanger sequencing (Additional file 1: Tables S1, S2). Finally, the pBS01 plasmid and the plasmids harboring cocoa genes were used to transform *S. cerevisiae* CEN.PK 113-11C, to construct 15 new yeast strains (Table 1).

## 20 ml shake flask cultivation and fatty acid analysis

20 ml shake flask fermentations were carried out in minimal medium (Verduyn et al. 1992) supplemented with 100 mg l^−1^ histidine. Three clones of each strain verified with PCR were used to inoculate 14 ml sterile falcon tubes containing 2 ml minimal medium and cultivated at 30 °C and 200 rpm for 24 h. The precultures were used to inoculate 100 ml shake flask with 20 ml fresh minimal medium at an initial OD600 nm of 0.1 that were then cultivated for 72 h at 30 °C and 200 rpm. The cultures were collected in 50 ml falcon tubes and harvested by centrifugation at 3000*g* for 10 min. The collected yeast cells were washed once with distilled water and dried using a freezer dryer (Alpha 2–4 LSC, Christ GmbH). Finally, 10 mg freeze-dried yeast biomass of each strain was used for fatty acid methyl ester (FAME) analyses using a microwave-assisted method (Khoomrung et al. 2012).

## 1 l shake flask batch cultivation and lipid analysis

In order to obtain enough lipids, 1 l yeast biomass of three selected yeast strains harboring cocoa genes and one yeast strain harboring pBS01A were collected and used for lipid extraction, respectively. Two clones of each strain verified by PCR were used to inoculate 20 ml fresh SC-URA medium in 50 ml falcon tubes and cultivated at 30 °C and 200 rpm for 24 h. Then the cultures of each strain were collected by centrifugation at 3000*g* for 10 min and resuspended in 50 ml fresh NLM medium. The resuspended yeast cells were used to inoculate 5 l shake flask with 1 l fresh NLM medium at an initial OD600 nm of 0.1, and cultivated at 30 °C and 200 rpm for 120 h. The yeast cells were harvested by centrifuging at 6000*g* for 15 min. 30 ml yeast cultures were collected separately and dried using a freezer dryer. The remaining yeast cells collected from 1 l yeast biomass were used for lipid extraction. The collected yeast cells were washed once with distilled water before further use. 10 mg of freeze dried yeast biomass was used for lipid extraction using a microwave-assisted methods followed by lipid analysis with HPLC-CAD (Khoomrung et al. 2013). The wet yeast biomass was used for large scale lipid extraction (Nambou et al. 2014; Yu et al. 2015). The lipid samples extracted from each strain were used for TAG analysis. TAGs in the lipids were measured by UPLC using RI detection, and the TAG compositions were expressed in relative area percentages (Shukla et al. 1983).

## Results

### Phylogenetic analysis of annotated cocoa GPAT, LPAT and DGAT genes revealed six potential CB biosynthetic genes

Usually, many TAG biosynthetic genes (GPAT, LPAT and DGAT genes) would be identified in one plant species, e.g. more than ten genes were annotated as GPAT genes in *T. cacao* (Argout et al. 2011; Motamayor et al. 2013). However, among all the cocoa genes annotated as GPAT, LPAT and DGAT genes, the ones actually responsible for CB biosynthesis are unknown (Argout et al. 2011; Motamayor et al. 2013). All amino acid sequences of annotated cocoa GPAT, LPAT and DGAT genes in the CGD and KEGG databases were downloaded and assigned the names TcGPAT1 to TcGPAT13, TcLPAT1 to TcLPAT10, TcDGAT1 to TcDGAT11, respectively. To identify potential CB biosynthetic genes, all annotated cocoa genes were compared with their corresponding reference genes of *A*. *thaliana*, *H. sapiens* and *S. cerevisiae* (Kanehisa et al. 2016). As GPAT, LPAT and DGAT genes of *S. cerevisiae* had been characterized and their function had been determined before, cocoa genes most similar to GPAT, LPAT and DGAT genes of *S. cerevisiae* might be functional in *S. cerevisiae* and they were prioritized for expression (Benghezal et al. 2007; Oelkers et al. 2002; Zheng and Zou 2001). In addition, genes which are similar to characterized GPAT, LPAT and DGAT genes of *A*. *thaliana* were also prioritized for expression.

**Fig. 3:**
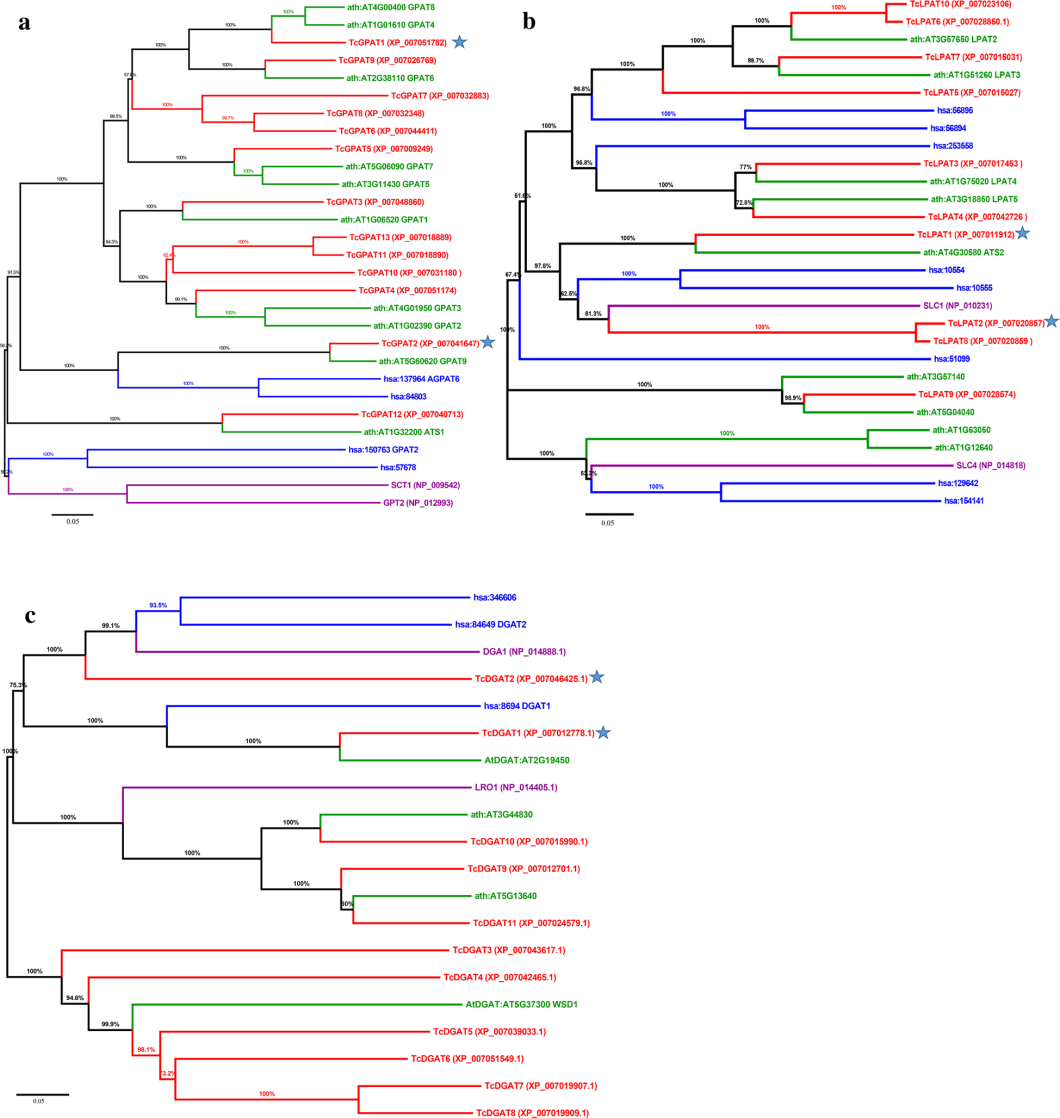
Phylogenetic analysis of GPAT (**a**), LPAT (**b**) and DGAT (**c**) genes of *T*. *cacao* genomes with an unrooted tree. All neighbor-joining trees were constructed using the MEGA 6.06 software (bootstrap values: 1000) with the peptide sequences. The sequences of *T*. *cacao* are labeled in* red*; the sequences of *A*. *thaliana* are labeled in *green*; the sequences of *S. cerevisiae* are labeled in *purple*; the sequences of *H. sapiens* are labeled in *blue*. The GPAT sequences of *T*. *cacao* were named TcGPAT1 to TcGPAT13; the LPAT sequences of *T*. *cacao* were named TcLPAT1 to TcLPAT10; the DGAT sequences of *T*. *cacao* were named TcDGAT1 to TcDGAT11. Cocoa genes selected for synthesis are marked with *asterisks*. The bootstrap values are marked above the nodes and the *scale bar* is indicated under each tree

Since all cocoa GPAT sequences were distinct from the two *S. cerevisiae* GPAT sequence of *SCT1* and *GPT2*, the potential cocoa CB biosynthetic GPAT sequences were selected by comparison with the GPAT sequences of *A*. *thaliana* (Fig. 3a). As TcGPAT12 has 63.9% identity with ATS1 of *A*. *thaliana*, which might not be involved in TAG biosynthetic pathway, it probably is not a potential CB biosynthetic GPAT gene (Chen et al. 2011; Nishida et al. 1993; Nuccio and Thomas 1999). GPAT1, -4, -5, -6 and -7 of *A*. *thaliana* had been demonstrated to have GPAT activity (Zheng et al. 2003), and GPAT4, -6 and -8 of *A*. *thaliana* strongly preferred C16:0 and C18:1 ω-oxidized acyl-CoAs over other substrates (Yang et al. 2012). TcGPAT1 has 81.8 and 82.8% identities with GPAT4 and GPAT8 of *A*. *thaliana*, respectively, and might be one potential CB biosynthetic GPAT gene (Fig. 3a). GPAT9 of *A*. *thaliana* was shown to be the ER-localized GPAT enzyme which is believed to be responsible for TAG biosynthesis (Shockey et al. 2016), and TcGPAT2 had 88.6% identity with GPAT9 of *A*. *thaliana* (Fig. 3a), suggesting *TcGPAT2* is one potential CB biosynthetic GPAT gene (Fig. 3a).

Though some annotated cocoa genes are very similar to the characterized LPAT genes of *A*. *thaliana*, three annotated cocoa LPAT sequences (TcLPAT1, TcLPAT2 and TcLPAT8) were very similar to the *S. cerevisiae* LPAT Slc1p (Benghezal et al. 2007; Nagiec et al. 1993), indicating these three cocoa genes might be the potential CB biosynthetic LPAT genes (Fig. 3b). As TcLPAT2 and TcLPAT8 have 95.5% identity, and consist of 310 and 200 amino acids, respectively, *TcLPAT2* is more likely to be a CB biosynthetic LPAT gene than *TcLPAT8*, because the most similar yeast LPAT sequence Slc1p has 303 amino acids and *TcLPAT8* might be a gene fragment of *TcLPAT2*.

For DGAT, two cocoa DGAT sequences, TcDGAT1 and TcDGAT2, are very similar to Dga1p of *S. cerevisiae* and a DGAT of *A*. *thaliana* (Katavic et al. 1995; Oelkers et al. 2002; Routaboul et al. 1999; Sorger and Daum 2002; Zou et al. 1999); while other cocoa DGAT sequences (TcDGAT3–TcDGAT11) are more similar to Lro1p of *S. cerevisiae* or wax ester synthase of *A*. *thaliana*, hinting that they might have DGAT activity, but not necessarily represent CB biosynthetic DGAT genes (Fig. 3c). Besides, TcDGAT1 and TcDGAT2 had been characterized before, the results indicated that they displayed DGAT activity. By combining the phylogenetic analysis and the enzyme activity analysis, it was hypothesized that *TcDGAT1* and *TcDGAT2* were the potential CB biosynthetic DGAT genes.

Two cocoa GPAT genes (*TcGPAT1* and *TcGPAT2*), two cocoa LPAT genes (*TcLPAT1* and *TcLPAT2*) and two cocoa DGAT genes (*TcDGAT1* and *TcDGAT2*), which are potentially responsible for CB biosynthesis, were therefore selected for codon optimization synthesis and expression in yeast based on this phylogenetic analyses.

### Expression of cocoa genes in *S. cerevisiae* changed its total fatty acid production

**Fig. 4:**
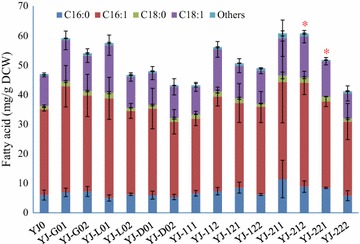
Total fatty acid production in different *S. cerevisiae* strains. Others represents the summed content of C12:0, C14:0, C14:1, C20:0, C20:1, C22:0, C24:0 and C26:0 fatty acids. The *error bars* represent the standard deviation of three biological replicates. *Asterisks* (*) indicate significant difference (p values are based on paired *t* tests corrected for multiple comparisons) between the yeast strains harboring cocoa genes and YJ0. *Asterisks* indicates p < 0.05; *Double Asterisks* indicates p < 0.01

The synthesized cocoa genes and cocoa gene combinations were assembled in expression cassettes using strong constitutive promoters and ligated into plasmid pBS01A, which resulted in 14 different plasmids (Additional file 1: Table S2; Fig. 2). Plasmid pBS01A and the 14 other plasmids harboring cocoa genes were introduced into *S. cerevisiae*, generating the control strain YJ0 and another 14 yeast strains, respectively (Table 1). The fatty acid production and composition of each yeast strains were measured after cultivation in shake flasks. The relative C16 and C18 content of all the 15 different yeast strains were more than 97.3%, which is consistent with previous studies that main fatty acids of *S. cerevisiae* are C16 and C18 (Khoomrung et al. 2012; Suutari et al. 1990). Compared with the control strain YJ0, fatty acid production of most yeast strains harboring cocoa genes increased (Fig. 4). Among the yeast strains harboring single cocoa genes, YJ-G01, YJ-G02 and YJ-L01 produced more total fatty acids than YJ0; YJ-D01 produced approximately the same amount of total fatty acids as YJ0; YJ-L02 and YJ-D01 produced less total fatty acids than YJ0. For the yeast strains harboring combinations of cocoa genes, YJ-112, -121, -122, -211, -212 and -221 produced more total fatty acids than YJ0, and YJ-111 and YJ-222 produced less total fatty acids than YJ0 (Fig. 4).

Due to variations between clones, among all the 14 yeast strains harboring cocoa genes, only YJ-212 (harboring the combination of *TcGPAT2*, *TcLPAT1* and *TcDGAT2*) and YJ-221 (harboring the combination of *TcGPAT2*, *TcLPAT2* and *TcDGAT1*) displayed a significant increase (29.1 and 9.9%, respectively) of the total fatty acid amount compared to YJ0 (Fig. 4). Moreover, C16:1, C18:0 and C18:1 contents of YJ-212, and C16:0, C18:0 and C18:1 contents of YJ-221 were increased compared with YJ0. The C16:1. C18:0 and C18:1 content of YJ-212 was 21.0, 54.0 and 36.2% higher compared with YJ0; and the C16:0, C18:0 and C18:1 content of YJ-221 was 41.2, 41.1 and 17.8% higher compared with YJ0 (Additional file 1: Table S3). In addition, the relative C16:0, C16:1, C18:0 and C18:1 contents of YJ-212 showed no significant difference compared with YJ0, but the relative C16:0, C16:1 and C18:0 contents of YJ-221 displayed a significant increase compared with the corresponding relative content of YJ0 (Additional file 1: Table S3). These results suggested that YJ-221 might be better for CBL production than YJ-212.

### Expression of cocoa gene combinations in *S. cerevisiae* altered lipid production and compositions

In order to further investigate the effects of cocoa gene expression on lipid and TAG production of *S. cerevisiae*, three yeast strains harboring cocoa genes, YJ-111, -121 and -221, and the control strain YJ0 were selected for lipid and TAG analyses. YJ-111 produced less fatty acids than YJ0; YJ-121 produced approximately the same amount of total fatty acids as YJ0; whereas YJ-221 produced more fatty acids than YJ0 and showed differences on C18:0 and C18:1 production and relative C18:0 content compared with YJ0 (Additional file 1: Table S3).

**Fig. 5:**
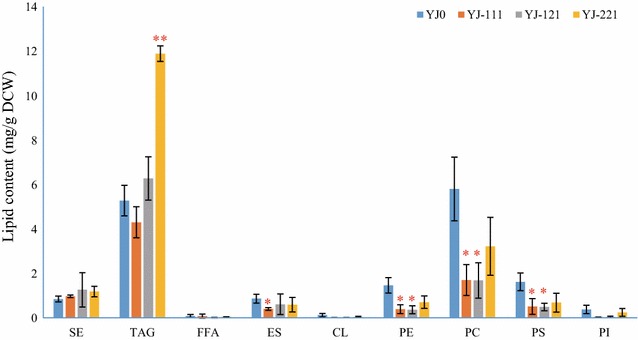
Total lipid production in different *S. cerevisiae* strains. *SE* steryl esters, *FFA* free fatty acids, *ES* ergosterol, *CL* cardiolipin, *PE* phosphatidylethanolamine, *PC* phosphatidylcholine, *PS* phosphatidylserine, *PI* phosphatidylinositol. Phosphatidic acids were not determined in this study. The *error bars* represent the standard deviation of two biological replicates. *Asterisks* (*) indicate significant difference (p values are based on paired *t* tests corrected for multiple comparisons) between the yeast strains harboring cocoa genes and YJ0. *Asterisks* indicates p < 0.05; *Double Asterisks* indicates p < 0.01

Total lipid profiles in yeasts usually cover TAGs, steryl esters (SE), ergosterol (ES), cardiolipin (CL), phosphatidic acid (PA), phosphatidylethanolamine (PE), phosphatidylinositol (PI), phosphatidylserine (PS), and phosphatidylcholine (PC) (Czabany et al. 2007; de Kroon et al. 2013; Kaneko et al. 1976). Though YJ-111 and YJ-121 did not display differences in total fatty acid production compared with YJ0 (Fig. 4), YJ-111 displayed differences compared with YJ0 in ES, PE, PC and PS production, YJ-121 showed differences compared with YJ0 in PE, PC and PS levels, suggesting the cocoa genes have an effect on yeast phospholipid production. YJ-221, which displayed differences with YJ0 in the total fatty acid content, also exhibited differences compared to YJ0 in TAG production (Fig. 5). In fact, YJ-221 produced 2.25-fold more TAG than YJ0. Though YJ-221 exhibited enhanced accumulation of TAGs, no other lipids of YJ-221 displayed differences compared with YJ0 (Fig. 5). Besides, while the TAG content comprised 37.2% of the total lipids in YJ0, TAGs represented 48.0, 56.3 and 60.3% of the total lipids in YJ-111, -121 and -221 (Fig. 5).

### Expressing cocoa gene combinations in *S. cerevisiae* increased potential CBL composition and production

**Fig. 6:**
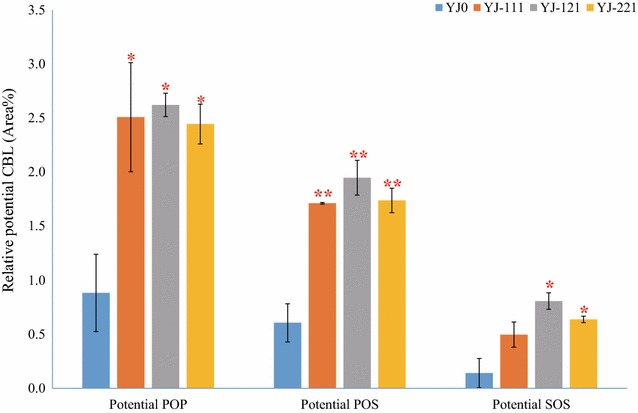
Relative potential CBL content of *S. cerevisiae* strains. The *error bars* represent the standard deviation of two biological replicates. *Asterisks* (*) indicate significant differences (p values are based on paired *t* tests corrected for multiple comparisons) between the yeast strains harboring cocoa genes and YJ0. *Asterisks* indicates p < 0.05; *Double Asterisks* indicates p < 0.01

Though the methods employed in this study do not allow to determine the exact position of each fatty acid within a TAG molecule, the fatty acid composition of each TAG can be determined. All four yeast strains tested in this study produced at least 22 different kinds of TAGs, and there was a major change in the TAG composition following expression of the cocoa genes (Fig. 6; Additional file 1: Figure S1). Most of the TAGs accounted for less than 5% of the total TAG pool (Additional file 1: Figure S1). Concerning CBL TAGs, potential POP (C16:0, C18:1, C16:0) and potential POS (C16:0, C18:1, C18:0), YJ-111, -121 and -221 displayed differences compared with YJ0; the proportion of potential POP in YJ-111, -121 and -221 increased by 185, 197 and 177%, respectively, while the proportion of potential POS increased by 183, 222 and 187%, respectively (Fig. 6). For another CBL TAG, potential SOS (C18:0, C18:1, C18:0), YJ-121 and YJ-221 also displayed a significant difference compared with YJ0. In fact, the potential SOS proportion had increased from 0.14% in YJ0 to 0.81% in YJ-121 and 0.64% in YJ-221, which means an increase of 476 and 354%, respectively (Fig. 6). The increase of potential CBL components, from 1.63% in YJ0 to 4.72% in YJ-111, 5.38% in YJ-121 and 4.82% in YJ-221 suggests that the cocoa genes are functional in *S. cerevisiae*. As YJ-221 also produces 2.25-fold more TAGs compared with YJ0, its potential CBL production was 6.7-fold improved compared with YJ0, showing that the combination of *TcGPAT2*, *TcLPAT2* and *TcDGAT1* (YJ-221) not only increased TAG production of *S. cerevisiae*, but also allowed *S. cerevisiae* to accumulate more potential CBL.

### Fatty acid profiles and compositions of the TAGs

**Fig. 7:**
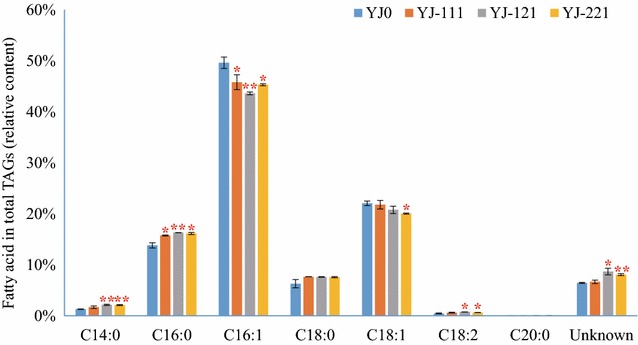
Relative fatty acid composition of the TAGs of each *S. cerevisiae* strain. The *error bars* represent the standard deviation of two biological replicates. *Asterisks* (*) indicate significant differences (p values are based on paired *t* tests corrected for multiple comparisons) between the yeast strains harboring cocoa genes and YJ0. *Asterisks* indicates p < 0.05; *Double Asterisks* indicates p < 0.01

In order to gain insight into substrate preferences of GPAT, LPAT and DGAT of *S. cerevisiae* under physiological conditions, the relative fatty acid composition of all TAGs was analyzed. The main fatty acids in the TAGs in these four yeast strains were C16 and C18 fatty acids, which is consistent with the total fatty acid composition results (Fig. 7). Generally, saturated fatty acids were increased in the TAGs of YJ-111, -121 and -221, which would be beneficial for CBL biosynthesis as there are more saturated fatty acids than unsaturated fatty acids in CB (Jahurul et al. 2013). In detail, the C16:0 proportion in TAGs of YJ-111, -121 and -221 was increased compared with YJ0, while the C16:1 proportion was reduced. Of the three engineered yeast strains, only YJ-221 exhibited a significant decrease in the C18:1 proportion. Also the C18:0 fatty acid ratio in the TAGs was increased for all the 3 yeast strain harboring cocoa genes, however, this increase was not significant, showing that it is necessary to screen additional cocoa GPAT, LPAT and DGAT genes in order to increase the incorporation of C18:0 into TAGs.

## Discussion

In this study, we aimed at increasing CBL production in *S. cerevisiae* by expressing genes of *T. cacao* potentially involved in CBL biosynthesis. There are at least 13 putative GPAT, 10 LPAT and 11 DGAT genes in *T*. *cacao*. By combining published information on the cocoa genome and a phylogenetic approach (Argout et al. 2011; Motamayor et al. 2013), we identified two GPAT, two LPAT and two DGAT genes of *T*. *cacao*, which might be responsible for CB biosynthesis. Expressing selected single cocoa genes or cocoa gene combinations (one GPAT, one LPAT and one DGAT gene) in *S. cerevisiae* had an effect on total fatty acid production in this study. Especially, expression of cocoa gene combinations was able to significantly alter the total fatty acid production in yeast, enabling some yeast strains to produce more CBL, indicating that the selected cocoa genes played important roles in CBL production in *S. cerevisiae*. As all the six cocoa genes were selected based on combining published information on the cocoa genome and a phylogenetic analysis, it is a clear example that phylogenetic analysis can be used for gene or pathway mining for metabolic engineering (Mak et al. 2015).

Though CBL production is determined by three different enzymes (Coleman and Lee 2004), expressing a single DGAT gene in *S. cerevisiae* can increase total TAG production (Bouvier-Navé et al. 2000; Runguphan and Keasling 2014). Besides, deletion or overexpression of GPAT or LPAT of *S. cerevisiae* can alter total lipid production and composition (Benghezal et al. 2007; Zheng and Zou 2001). By comparing the three engineered yeast strains YJ-111, YJ-121 and YJ-221 with YJ0, we showed that lipid and TAG composition of these three yeast strains harboring cocoa genes were different from YJ0, suggesting expression of some GPAT, LPAT and DGAT genes could increase yeast TAG production and the cocoa gene combinations functioned in *S. cerevisiae*. The potential CBL production of the three yeast strains, especially, potential SOS production drastically increased, indicating the selected cocoa genes are promising candidate CB biosynthetic genes.

Compared with the fact that there are 14–16.4% POP, 34.6–38.3% POS and 23.7–28.4% SOS in CB (Lipp and Anklam 1998), the CBL content in the engineered *S. cerevisiae* strains obtained in this study is less than 5.4%, demonstrating that more efforts are need to improve CBL production in yeast for further industrial application. Considering that YJ-221 was the only of the three yeast strains that showed significant difference with YJ0 in the C18:1 proportion of TAGs and that the remaining 11 cocoa GPAT and 8 cocoa LPAT genes not selected in this study might also have effects on CBL biosynthesis (Argout et al. 2011; Motamayor et al. 2013), suggests that expressing some of these in *S. cerevisiae* might improve CBL production and reveal more candidate genes for yeast CBL production. In addition, many strategies have been implemented for increasing fatty acid or TAG production, such as overexpression of fatty acid or TAG biosynthesis genes of DGA1, acetyl-CoA carboxylase, fatty acid synthase (Kamisaka et al. 2007; Runguphan and Keasling 2014; Zhou et al. 2016), and these could also be used for further increasing CBL production in *S. cerevisiae*.

In conclusion, we increased CBL production by *S. cerevisiae* through expressing selected genes of *T. cacao* potentially involved in CB biosynthesis, which might be used in yeast CBL production in future. Additionally, our approach of integrating plant genome data screening and metabolic engineering may also find application in production of other value-added plant metabolites using *S. cerevisiae* as a cell factory.

## Additional file

**Additional file 1.** Additional tables and figure.

## Abbreviations

CB: cocoa butter
TAG: triacylglycerol
POP: 1,3-dipalmitoyl-2-oleoyl-glycerol (C16:0-C18:1-C16:0)
POS: 1-palmitoyl-3-stearoyl-2-oleoyl-glycerol (C16:0-C18:1-C18:0)
SOS: 1,3-distearoyl-2-oleoyl-glycerol (C18:0-C18:1-C18:0)
CBL: CB-like lipids
GPAT: glycerol-3-phosphate acyltransferase
LPAT: lysophospholipid acyltransferase
DGAT: diacylglycerol acyltransferase
acyl-CoA: acyl-coenzyme A
DAG: diacylglycerol
CG: Cacao Genome Database
SE: steryl esters
ES: ergosterol
CL: cardiolipin
PA: phosphatidic acid
PE: phosphatidylethanolamine
PI: phosphatidylinositol
PS: phosphatidylserine
PC: phosphatidylcholine
