# Supplementary material for: Increasing cocoa butter-like lipid production of Saccharomyces cerevisiae by expression of selected cocoa genes
Source: AMB Express. 2017 Feb 6;7:34. doi: 10.1186/s13568-017-0333-1 (PMC5293708; doi:10.1186/s13568-017-0333-1)
Supplement: Supplementary file 1 — Additional file 1. Additional tables and figure. [file 13568_2017_333_MOESM1_ESM.docx]

**Increasing cocoa butter-like lipid production of *Saccharomyces cerevisiae* by expression of selected cocoa genes**

**Yongjun Wei^1,2^ · Michael Gossing^1,2^ · David Bergenholm^1,2^ · Verena Siewers^1,2^ · Jens Nielsen^1,2,3^**

**^1^** Department of Biology and Biological Engineering, Chalmers University of Technology, SE-41296 Gothenburg, Sweden

**^2^** Novo Nordisk Foundation Center for Biosustainability, Chalmers University of Technology, SE-41296 Gothenburg, Sweden

**^3^** Novo Nordisk Foundation Center for Biosustainability, Technical University of Denmark, DK-2800 Kgs. Lyngby, Denmark

**E-mail:**

Yongjun Wei: [weyongjun@163.com](mailto:weyongjun@163.com)

Michael Gossing: gossing@chalmers.se

David Bergenholm: davidju@chalmers.se

Verena Siewers: siewers@chalmers.se

Jens Nielsen: [nielsenj@chalmers.se](mailto:nielsenj@chalmers.se)

**Corresponding author**

Jens Nielsen

E-mail: [nielsenj@chalmers.se](mailto:nielsenj@chalmers.se)

Telephone: +46 (0)31 772 3804

Fax: +46(0)31 772 3801

Number of pages: 28 (including cover page)

Number of figures and tables: 4

**
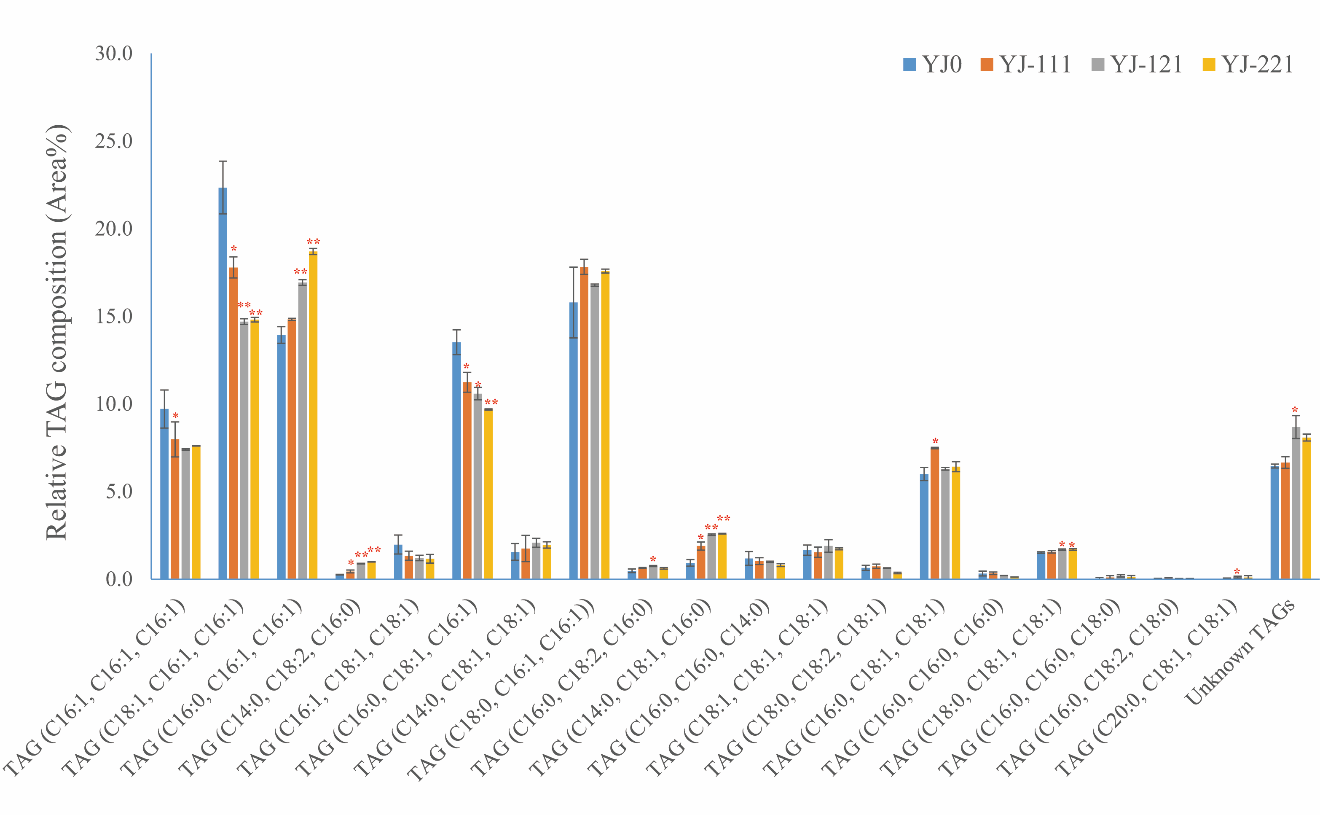
**

Fig. S1, Relative TAG content (except potential CBL) of *S. cerevisiae* strains. The error bars represent the standard deviation of two biological replicates. Asterisks (*) indicate significant differences (p-values are based on paired t-tests corrected for multiple comparisons) between the yeast strains harboring cocoa genes and YJ0. * indicates p<0.05; ** indicates p<0.01.

**Table S1 List of primers used in this study**

| Name | Sequence(5'-3') | Application |
| --- | --- | --- |
| P*_TEF1_*-SGE-fw | CAGTTATTACCCGCGATCGCGACATGGAGGCCCAGAATAC | *TEF1* promoter amplification and fusion to pBS01A fragment (only for single GPAT gene expression); PCR verification |
| P*_TEF1_*-rv | GGTTGTTTATGTTCGGATGTGATG | *TEF1* promoter amplification |
| TcGPAT1-fw | TTCTCACATCACATCCGAACATAAACAACCATGGCTCCAGCTAAATCTGGTAG | *TcGPAT1* amplification and fusion to *TEF1* promoter |
| TcGPAT1-rv | AATAAAAATCATAAATCATAAGAAATTCGCTTACTTCTTGGCGTTGTACATAGAT | *TcGPAT1* amplification and fusionfusion to *ADH1* terminator |
| TcGPAT2-fw | TTCTCACATCACATCCGAACATAAACAACCATGAAGAAAGAAAAGTTGTTCGGT | *TcGPAT2* amplification and fusionfusion to *TEF1* promoter |
| TcGPAT2-rv | AATAAAAATCATAAATCATAAGAAATTCGCTCACTTTTCTTCCAATCTCAACA | *TcGPAT2* amplification and fusionfusion to *ADH1* terminator |
| T*_ADH1_*-fw | GCGAATTTCTTATGATTTATGATTTTTAT | *ADH1* terminator amplification |
| T*_ADH1_*-SGE-rv | CTAACAACAACAACCTCGAGTTGCTCGGCATGCCGGTAGAG | *ADH1* terminator amplification and fusionfusion to pBS01A plasmid (only one single GPAT gene expression); PCR verification |
| P*_PGK1_*-SGE-fw | CAGTTATTACCCGCGATCGCCTGGAAGTACCTTCAAAGAATG | *PGK1* promoter amplification and fusionfusion to pBS01A plasmid (only one single LPAT gene expression); PCR verification |
| P*_PGK1_*-rv | TGTTTTATATTTGTTGTAAAAAGTAGATAAT | *PGK1* promoter amplification |
| TcLPAT1-fw | TTATCTACTTTTTACAACAAATATAAAACAATGGAATTGTCATCCTTGCCATCC | *TcLPAT1* amplification and fusion to *PGK1* promoter |
| TcLPAT1-rv | ATGGATATTATATGAATGTATGATTTTATATTAACATTGATGTTTCAAGGTATCGGC | *TcLPAT1* amplification and fusion to *GAT2* terminator |
| TcLPAT2-fw | TTATCTACTTTTTACAACAAATATAAAACAATGGAATCTTCAGGTTCTGGTTCAT | *TcLPAT2* amplification and fusion to *PGK1* promoter |
| TcLPAT2-rv | ATGGATATTATATGAATGTATGATTTTATATCAAGATCTAGAGGAGTTAGTGGTA | *TcLPAT2* amplification and fusion to *GAT2* terminator |
| T*_GAT2_*-fw | TATAAAATCATACATTCATATAATATCCAT | *GAT2* terminator amplification |
| T*_GAT2_*-SGE-rv | CTAACAACAACAACCTCGAGGGGAAAACGTTAGGAAAACG | *GAT2* terminator amplification and fusion to pBS01A fragment (only one single LPAT gene expression); PCR verification |
| P*_FBA1_*-SGE-fw | CAGTTATTACCCGCGATCGCCACTGGTAGAGAGCGACTTTG | *FBA1* promoter amplification and fusion to pBS01A fragment (only one single DGAT gene expression); PCR verification |
| P*_FBA1_*-rv | TTTGAATATGTATTACTTGGTTATGGTT | *FBA1* promoter amplification |
| TcDGAT1-fw | ATAACCATAACCAAGTAATACATATTCAAAATGGCCATTTCTGATTCCCCAG | *TcDGAT1* amplification and fusion to *FBA1* promoter |
| TcDGAT1-rv | CATGATATCGACAAAGGAAAAGGGGCCTGTTCAATCGGCCTTACCCTTTCTGT | *TcDGAT1* amplification and fusion to *CYC1* terminator |
| TcDGAT2-fw | ATAACCATAACCAAGTAATACATATTCAAAATGATGGGTGAAGAAATGGAAG | *TcDGAT2* amplification and fusion to *FBA1* promoter |
| TcDGAT2-rv | CATGATATCGACAAAGGAAAAGGGGCCTGTTCACAAGATCTTCAATGGCAAATC | *TcDGAT2* amplification and fusion to *CYC1* terminator |
| T*_CYC1_*-fw | ACAGGCCCCTTTTCCTTTGT | *CYC1* terminator amplification |
| T*_CYC1-SGE_*-rv | CTAACAACAACAACCTCGAGTTTGTACAGAAAAAAAAGAAAAATTTGAAAT | DGAT gene expression cassette amplification, *CYC1* terminator amplification and fusion to pBS01A fragment (one single DGAT gene expression or 3 genes of GPAT, LPAT and DGAT expression); PCR verification |
| pBS01A-fw | CTCGAGGTTGTTGTTGTTAGATCT | Backbone fragment of plasmid pBS01A amplification. |
| pBS01A-rv | GCGATCGCGGGTAATAACTGAT | Backbone fragment of plasmid pBS01A amplification. |
| T*_ADH1_*-3GE-rv | CAGTTATTACCCGCGATCGCTTGCTCGGCATGCCGGTAGAG | GPAT gene expression cassette amplification, *ADH1* terminator amplification and fusion to pBS01A fragment (3 genes of GPAT, LPAT and DGAT expression) |
| P*_TEF1_*-3GE-fw | TGAAGGTACTTCCAGGACATGGAGGCCCAGAATAC | GPAT gene expression cassette amplification and fusion to *TEF1* promoter and *PGK1* promoter |
| P*_PGK1_*-SGE-fw | CTGGGCCTCCATGTCCTGGAAGTACCTTCAAAGAATG | LPAT gene expression cassette amplification and fusion to *TEF1* promoter and *PGK1* promoter |
| T*_GAT2_*-3GE-rv | CGCTCTCTACCAGTGGGGAAAACGTTAGGAAAACG | LPAT gene expression cassette amplification and fusion to *GAT2* terminator and *FBA1* promoter |
| P*_FBA1_*-3GE-fw | TCCTAACGTTTTCCCCACTGGTAGAGAGCGACTTTG | DGAT gene expression cassette amplification and fusion to *GAT2* terminator and *FBA1* promoter |
| P*_TEF1_*-verificatoin-fw | GACATGGAGGCCCAGAATAC | Sequencing primers for expression cassette verification |
| P*_PGK1_*-verificatoin-fw | GATGCTTTCTTTTTCTCTTTTTTAC | Sequencing primers for expression cassette verification |
| P*_FBA1_*-verificatoin-fw | TTCTCTTCTGACTTTGACTC | Sequencing primers for expression cassette verification |

**Table S2 List of plasmids constructed in this study.**

| Name | Parent plasmid | Properties | Reference |
| --- | --- | --- | --- |
| pBS01A | pSP-GM1 | Expression empty plasmid, *amp* (ampicillin resistance), *URA3* | Provided by Anastasia Krivoruchko |
| pYJ-G01 | pBS01A | Expression plasmid with gene of TcGPAT01 | This study |
| pYJ-G02 | pBS01A | Expression plasmid with gene of TcGPAT02 | This study |
| pYJ-L01 | pBS01A | Expression plasmid with gene of TcLPAT01 | This study |
| pYJ-L02 | pBS01A | Expression plasmid with gene of TcLPAT02 | This study |
| pYJ-D01 | pBS01A | Expression plasmid with gene of TcDGAT01 | This study |
| pYJ-D02 | pBS01A | Expression plasmid with gene of TcDGAT02 | This study |
| pYJ-111 | pBS01A | Expression plasmid with genes of TcGPAT01, TcLPAT01, TcDGAT01 | This study |
| pYJ-112 | pBS01A | Expression plasmid with genes of TcGPAT01, TcLPAT01, TcDGAT02 | This study |
| pYJ-121 | pBS01A | Expression plasmid with genes of TcGPAT01, TcLPAT02, TcDGAT01 | This study |
| pYJ-122 | pBS01A | Expression plasmid with genes of TcGPAT01, TcLPAT02, TcDGAT02 | This study |
| pYJ-211 | pBS01A | Expression plasmid with genes of TcGPAT02, TcLPAT01, TcDGAT01 | This study |
| pYJ-212 | pBS01A | Expression plasmid with genes of TcGPAT02, TcLPAT01, TcDGAT02 | This study |
| pYJ-221 | pBS01A | Expression plasmid with genes of TcGPAT02, TcLPAT02, TcDGAT01 | This study |
| pYJ-222 | pBS01A | Expression plasmid with genes of TcGPAT02, TcLPAT02, TcDGAT02 | This study |

**Table S3 Total fatty acid composition and standard deviation of 15 yeast strains (n=3)**

|  | Fatty acids (mg/g DCW) | | | | |
| --- | --- | --- | --- | --- | --- |
|  | C16:0 | C16:1 | C18:0 | C18:1 | Others |
| YJ0 | 5.98 ± 1.67 | 29.06 ± 0.61 | 1.16 ± 0.32 | 10.18 ± 0.35 | 0.62 ± 0.35 |
| YJ-G01 | 6.92 ± 1.41 | 35.94 ± 6.99 | 1.60 ± 0.33 | 13.94 ± 3.15 | 0.64 ± 0.09 |
| YJ-G02 | 7.24 ± 1.68 | 32.53 ± 7.15 | 1.45 ± 0.23 | 12.00 ± 2.31 | 0.74 ± 0.23 |
| YJ-L01 | 5.03 ± 1.05 | 33.72 ± 7.12 | 1.94 ± 0.33 | 16.05 ± 3.43 | 0.74 ± 0.22 |
| YJ-L02 | 6.22 ± 0.41 | 28.29 ± 2.52 | 1.02 ± 0.07 | 10.45 ± 1.42 | 0.64 ± 0.15 |
| YJ-D01 | 5.94 ± 1.34 | 29.36 ± 6.88 | 1.20 ± 0.28 | 10.80 ± 2.22 | 0.59 ± 0.17 |
| YJ-D02 | 5.32 ± 0.92 | 25.47 ± 4.13 | 1.24 ± 0.31 | 10.63 ± 2.75 | 0.46 ± 0.07 |
| YJ-111 | 6.59 ± 0.97 | 25.32 ± 2.44 | 1.39 ± 0.03 | 9.31 ± 1.52 | 0.54 ± 0.58 |
| YJ-112 | 7.31 ± 1.28 | 32.05 ± 3.20 | 1.68 ± 0.45 | 14.27 ± 2.66 | 0.74 ± 0.20 |
| YJ-121 | 8.50 ± 1.86 | 28.68 ± 6.59 | 1.58 ± 0.36 | 11.05 ± 2.35 | 0.84 ± 0.06 |
| YJ-122 | 6.16 ± 0.35 | 29.66 ± 5.23 | 1.14 ± 0.08 | 11.15 ± 1.04 | 0.81 ± 0.18 |
| YJ-211 | 11.41 ± 6.37 | 32.90 ± 13.85 | 2.20 ± 1.16 | 12.60 ± 6.16 | 1.61 ± 0.88 |
| YJ-212 | 8.83 ± 1.93 | 35.16 ± 3.94 | 1.79 ± 0.36 | 13.87 ± 2.11 | 1.03 ± 0.20 |
| YJ-221 | 8.45 ± 0.24 | 29.26 ± 1.75 | 1.64 ± 0.01 | 11.98 ± 1.24 | 0.37 ± 0.09 |
| YJ-222 | 5.79 ± 1.68 | 25.10 ± 6.09 | 0.97 ± 0.30 | 8.57 ± 2.55 | 0.75 ± 0.12 |

**pBS01A plasmid annotation**

| Feature name | Start | End |
| --- | --- | --- |
| URA3 | 9 | 1106 |
| T*_ADH1_* | 1115 | 1314 |
| P*_TEF1_* | 1385 | 1805 |
| P*_PGK1_* | 1812 | 2796 |
| T*_CYC1_* | 2857 | 3054 |
| pUC origin | 3116 | 3784 |
| Ampicillin resistance gene | 3785 | 4778 |
| 2-µm origin | 4779 | 5934 |

**pBS01A Sequence**

1 ggcgcgcctt caattcatca tttttttttt attctttttt ttgatttcgg tttctttgaa

61 atttttttga ttcggtaatc tccgaacaga aggaagaacg aaggaaggag cacagactta

121 gattggtata tatacgcata tgtagtgttg aagaaacatg aaattgccca gtattcttaa

181 cccaactgca cagaacaaaa acctgcagga aacgaagata aatcatgtcg aaagctacat

241 ataaggaacg tgctgctact catcctagtc ctgttgctgc caagctattt aatatcatgc

301 acgaaaagca aacaaacttg tgtgcttcat tggatgttcg taccaccaag gaattactgg

361 agttagttga agcattaggt cccaaaattt gtttactaaa aacacatgtg gatatcttga

421 ctgatttttc catggagggc acagttaagc cgctaaaggc attatccgcc aagtacaatt

481 ttttactctt cgaagacaga aaatttgctg acattggtaa tacagtcaaa ttgcagtact

541 ctgcgggtgt atacagaata gcagaatggg cagacattac gaatgcacac ggtgtggtgg

601 gcccaggtat tgttagcggt ttgaagcagg cggcagaaga agtaacaaag gaacctagag

661 gccttttgat gttagcagaa ttgtcatgca agggctccct atctactgga gaatatacta

721 agggtactgt tgacattgcg aagagcgaca aagattttgt tatcggcttt attgctcaaa

781 gagacatggg tggaagagat gaaggttacg attggttgat tatgacaccc ggtgtgggtt

841 tagatgacaa gggagacgca ttgggtcaac agtatagaac cgtggatgat gtggtctcta

901 caggatctga cattattatt gttggaagag gactatttgc aaagggaagg gatgctaagg

961 tagagggtga acgttacaga aaagcaggct gggaagcata tttgagaaga tgcggccagc

1021 aaaactaaaa aactgtatta taagtaaatg catgtatact aaactcacaa attagagctt

1081 caatttaatt atatcagtta ttacccgcga tcgcttgctc ggcatgccgg tagaggtgtg

1141 gtcaataaga gcgacctcat gctatacctg agaaagcaac ctgacctaca ggaaagagtt

1201 actcaagaat aagaattttc gttttaaaac ctaagagtca ctttaaaatt tgtatacact

1261 tatttttttt ataacttatt taataataaa aatcataaat cataagaaat tcgcgcggcc

1321 gcgttgttcc tagggttgtt gaattcgttg ttgagctcgt tgttttaatt aagttgttac

1381 tagtttgtaa ttaaaactta gattagattg ctatgctttc tttctaatga gcaagaagta

1441 aaaaaagttg taatagaaca agaaaaatga aactgaaact tgagaaattg aagaccgttt

1501 attaacttaa atatcaatgg gaggtcatcg aaagagaaaa aaatcaaaaa aaaaaatttt

1561 caagaaaaag aaacgtgata aaaattttta ttgccttttt cgacgaagaa aaagaaacga

1621 ggcggtctct tttttctttt ccaaaccttt agtacgggta attaacgaca ccctagagga

1681 agaaagaggg gaaatttagt atgctgtgct tgggtgtttt gaagtggtac ggcgatgcgc

1741 ggagtccgag aaaatctgga agagtaaaaa aggagtagaa acattttgaa gctatggtgt

1801 gtgcggccgg cctggaagta ccttcaaaga atggggtctt atcttgtttt gcaagtacca

1861 ctgagcagga taataataga aatgataata tactatagta gagataacgt cgatgacttc

1921 ccatactgta attgctttta gttgtgtatt tttagtgtgc aagtttctgt aaatcgatta

1981 attttttttt ctttcctctt tttattaacc ttaattttta ttttagattc ctgacttcaa

2041 ctcaagacgc acagatatta taacatctgc ataataggca tttgcaagaa ttactcgtga

2101 gtaaggaaag agtgaggaac tatcgcatac ctgcatttaa agatgccgat ttgggcgcga

2161 atcctttatt ttggcttcac cctcatacta ttatcagggc cagaaaaagg aagtgtttcc

2221 ctccttcttg aattgatgtt accctcataa agcacgtggc ctcttatcga gaaagaaatt

2281 accgtcgctc gtgatttgtt tgcaaaaaga acaaaactga aaaaacccag acacgctcga

2341 cttcctgtct tcctattgat tgcagcttcc aatttcgtca cacaacaagg tcctagcgac

2401 ggctcacagg ttttgtaaca agcaatcgaa ggttctggaa tggcgggaaa gggtttagta

2461 ccacatgcta tgatgcccac tgtgatctcc agagcaaagt tcgttcgatc gtactgttac

2521 tctctctctt tcaaacagaa ttgtccgaat cgtgtgacaa caacagcctg ttctcacaca

2581 ctcttttctt ctaaccaagg gggtggttta gtttagtaga acctcgtgaa acttacattt

2641 acatatatat aaacttgcat aaattggtca atgcaagaaa tacatatttg gtcttttcta

2701 attcgtagtt tttcaagttc ttagatgctt tctttttctc ttttttacag atcatcaagg

2761 aagtaattat ctacttttta caacaaatat aaaacaggat ccgttgttgt tgttgtcgac

2821 gttgttgcta gcgttgttgg taccgttgtt caattgacag gccccttttc ctttgtcgat

2881 atcatgtaat tagttatgtc acgcttacat tcacgccctc cccccacatc cgctctaacc

2941 gaaaaggaag gagttagaca acctgaagtc taggtcccta tttatttttt tatagttatg

3001 ttagtattaa gaacgttatt tatatttcaa atttttcttt tttttctgta caaactcgag

3061 gttgttgttg ttagatctgt tgttgttgtt tccggagttg ttgttgttcg ccggcgtgag

3121 caaaaggcca gcaaaaggcc aggaaccgta aaaaggccgc gttgctggcg tttttccata

3181 ggctccgccc ccctgacgag catcacaaaa atcgacgctc aagtcagagg tggcgaaacc

3241 cgacaggact ataaagatac caggcgtttc cccctggaag ctccctcgtg cgctctcctg

3301 ttccgaccct gccgcttacc ggatacctgt ccgcctttct cccttcggga agcgtggcgc

3361 tttctcatag ctcacgctgt aggtatctca gttcggtgta ggtcgttcgc tccaagctgg

3421 gctgtgtgca cgaacccccc gttcagcccg accgctgcgc cttatccggt aactatcgtc

3481 ttgagtccaa cccggtaaga cacgacttat cgccactggc agcagccact ggtaacagga

3541 ttagcagagc gaggtatgta ggcggtgcta cagagttctt gaagtggtgg cctaactacg

3601 gctacactag aagaacagta tttggtatct gcgctctgct gaagccagtt accttcggaa

3661 aaagagttgg tagctcttga tccggcaaac aaaccaccgc tggtagcggt ggtttttttg

3721 tttgcaagca gcagattacg cgcagaaaaa aaggatctca agaagatcct ttgatctttt

3781 ctacttacca atgcttaatc agtgaggcac ctatctcagc gatctgtcta tttcgttcat

3841 ccatagttgc ctgactcccc gtcgtgtaga taactacgat acgggagggc ttaccatctg

3901 gccccagtgc tgcaatgata ccgcgagacc cacgctcacc ggctccagat ttatcagcaa

3961 taaaccagcc agccggaagg gccgagcgca gaagtggtcc tgcaacttta tccgcctcca

4021 tccagtctat taattgttgc cgggaagcta gagtaagtag ttcgccagtt aatagtttgc

4081 gcaacgttgt tgccattgct acaggcatcg tggtgtcacg ctcgtcgttt ggtatggctt

4141 cattcagctc cggttcccaa cgatcaaggc gagttacatg atcccccatg ttgtgcaaaa

4201 aagcggttag ctccttcggt cctccgatcg ttgtcagaag taagttggcc gcagtgttat

4261 cactcatggt tatggcagca ctgcataatt ctcttactgt catgccatcc gtaagatgct

4321 tttctgtgac tggtgagtac tcaaccaagt cattctgaga atagtgtatg cggcgaccga

4381 gttgctcttg cccggcgtca atacgggata ataccgcgcc acatagcaga actttaaaag

4441 tgctcatcat tggaaaacgt tcttcggggc gaaaactctc aaggatctta ccgctgttga

4501 gatccagttc gatgtaaccc actcgtgcac ccaactgatc ttcagcatct tttactttca

4561 ccagcgtttc tgggtgagca aaaacaggaa ggcaaaatgc cgcaaaaaag ggaataaggg

4621 cgacacggaa atgttgaata ctcatactct tcctttttca atattattga agcatttatc

4681 agggttattg tctcatgagc ggatacatat ttgaatgtat ttagaaaaat aaacaaatag

4741 gggttccgcg cacatttccc cgaaaagtgc cacctgaacg aagcatctgt gcttcatttt

4801 gtagaacaaa aatgcaacgc gagagcgcta atttttcaaa caaagaatct gagctgcatt

4861 tttacagaac agaaatgcaa cgcgaaagcg ctattttacc aacgaagaat ctgtgcttca

4921 tttttgtaaa acaaaaatgc aacgcgagag cgctaatttt tcaaacaaag aatctgagct

4981 gcatttttac agaacagaaa tgcaacgcga gagcgctatt ttaccaacaa agaatctata

5041 cttctttttt gttctacaaa aatgcatccc gagagcgcta tttttctaac aaagcatctt

5101 agaatacttt ttttctcctt tgtgcgctct ataatgcagt ctcttgataa ctttttgcac

5161 tgtaggtccg ttaaggttag aagaaggcta ctttggtgtc tattttctct tccataaaaa

5221 aagcctgact ccacttcccg cgtttactga ttactagcga agctgcgggt gcattttttc

5281 aagataaagg catccccgat tatattctat accgatgtgg attgcgcata ctttgtgaac

5341 agaaagtgat agcgttgatg attcttcatt ggtcagaaaa ttatgaacgg tttcttctat

5401 tttgtctcta tatactacgt ataggaaatg tttacatttt cgtattgttt tcgattcact

5461 ctatgaatag ttcttactac aatttttttg tctaaagagt aatactagag ataaacataa

5521 aaaatgtaga ggtcgagttt agatgcaagt tcaaggagcg aaaggtggat gggtaggtta

5581 tatagggata tagcacagag atatatagca aagagatact tttgagcaat gtttgtggaa

5641 gcggtattcg caatatttta gtagctcgtt acagtccggt gcgtttttgg ttttttgaaa

5701 gtgcgtcttc agagcgcttt tggttttcaa aagcgctctg aagttcctat actttctaga

5761 gaataggaac ttcggaatag gaacttcaaa gcgtttccga aaacgagcgc ttccgaaaat

5821 gcaacgcgag ctgcgcacat acagctcact gttcacgtcg cacctatatc tgcgtgttgc

5881 ctgtatatat atatacatga gaagaacggc atagtgcgtg tttatgctta aatg

//

**GPAT genes used for phylogenetic tree construction of Fig 3a.**

>ath:AT5G60620 GPAT9

MSSTAGRLVTSKSELDLDHPNIEDYLPSGSSINEPRGKLSLRDLLDISPTLTEAAGAIVDDSFTRCFKSNPPEPWNWNIYLFPLYCFGVVVRYCILFPLRCFTLAFGWIIFLSLFIPVNALLKGQDRLRKKIERVLVEMICSFFVASWTGVVKYHGPRPSIRPKQVYVANHTSMIDFIVLEQMTAFAVIMQKHPGWVGLLQSTILESVGCIWFNRSEAKDREIVAKKLRDHVQGADSNPLLIFPEGTCVNNNYTVMFKKGAFELDCTVCPIAIKYNKIFVDAFWNSRKQSFTMHLLQLMTSWAVVCEVWYLEPQTIRPGETGIEFAERVRDMISLRAGLKKVPWDGYLKYSRPSPKHSERKQQSFAESILARLEEK

>TcGPAT2(XP_007041647)

MSSRGGKLSSSSSELDLDGPNIEDYLPSGSSINEPRGKLRLRDLLDISPTLTEAAGAIVDDSFTRCFKSNPPEPWNWNVYLFPLWCFGVAVRYLILFPARVVVLTIGWIIFLSSFIPVHFLLKGHDKLRKKMERVLVELMCSFFVASWTGVVKYHGPRPSIRPKQVFVANHTSMIDFIILEQMTAFAVIMQKHPGWVGLLQSTILESVGCIWFNRSEAKDREIVAKKLRDHVQGVDNNPLLIFPEGTCINNQYSVMFKKGAFELGCTVCPIAIKYNKIFVDAFWNSRKQSFTMHLLQLMTSWAVVCDVWYLEPQNLRPGETPIEFAERVRDIISVRAGLKKVPWDGYLKYSRPSPKHRERKQQSFAESVLLRLEEK

>hsa:84803

MEGAELAGKILSTWLTLVLGFILLPSVFGVSLGISEIYMKILVKTLEWATIRIEKGTPKESILKNSASVGIIQRDESPMEKGLSGLRGRDFELSDVFYFSKKGLEAIVEDEVTQRFSSEELVSWNLLTRTNVNFQYISLRLTMVWVLGVIVRYCVLLPLRVTLAFIGISLLVIGTTLVGQLPDSSLKNWLSELVHLTCCRICVRALSGTIHYHNKQYRPQKGGICVANHTSPIDVLILTTDGCYAMVGQVHGGLMGIIQRAMVKACPHVWFERSEMKDRHLVTKRLKEHIADKKKLPILIFPEGTCINNTSVMMFKKGSFEIGGTIHPVAIKYNPQFGDAFWNSSKYNMVSYLLRMMTSWAIVCDVWYMPPMTREEGEDAVQFANRVKSAIAIQGGLTELPWDGGLKRAKVKDIFKEEQQKNYSKMIVGNGSLS

>TcGPAT7(XP_007032883)

MGSRILPSASLFLCSVFLSSFCASCRSSLSIPPLRASFPCASSKSLAPICRLPLPHACGLGILVLFTASSLAMADVVADVVAPVKPKNRASKKFSSRGVDLDVLLEMSSSNSSPLLSPKVFLRDLKRRPLALTKKILSWALEGARSTAFSLGTGGKFHYPARREFFASGRLLFLLLASPLAGTLYYFTSESHDIRVLIFATFVGMKVSDIESVARAVLPKFYSSDLHPGFRFMWEEMCAYGDSEGHSGVKSPGVLVGKNKADALENESHVVPAKAEAGAVSHDKLPKPIVFHDGRLVQNPTPFMALLTILWIPVGFLLACLRIAVGILLPTPLVYYAMWPLGFRIHIKGTPPQPTKKSIGQTVVLFACSHRTLLDPIALSVALCRPIPTATYSISGLSEIISPIKTARLCLDRATDASIIKKLLEKGDLVICPEGTKT

>hsa:137964 AGPAT6

MFLLLPFDSLIVNLLGISLTVLFTLLLVFIIVPAIFGVSFGIRKLYMKSLLKIFAWATLRMERGAKEKNHQLYKPYTNGIIAKDPTSLEEEIKEIRRSGSSKALDNTPEFELSDIFYFCRKGMETIMDDEVTKRFSAEELESWNLLSRTNYNFQYISLRLTVLWGLGVLIRYCFLLPLRIALAFTGISLLVVGTTVVGYLPNGRFKEFMSKHVHLMCYRICVRALTAIITYHDRENRPRNGGICVANHTSPIDVIILASDGYYAMVGQVHGGLMGVIQRAMVKACPHVWFERSEVKDRHLVAKRLTEHVQDKSKLPILIFPEGTCINNTSVMMFKKGSFEIGATVYPVAIKYDPQFGDAFWNSSKYGMVTYLLRMMTSWAIVCSVWYLPPMTREADEDAVQFANRVKSAIARQGGLVDLLWDGGLKREKVKDTFKEEQQKLYSKMIVGNHKDRSRS

>ath:AT1G32200 ATS1

MTLTFSSSAATVAVAAATVTSSARVPVYPLASSTLRGLVSFRLTAKKLFLPPLRSRGGVSVRAMSELVQDKESSVAASIAFNEAAGETPSELSHSRTFLDARSEQDLLSGIKKEAEAGRLPANVAAGMEELYWNYKNAVLSSGASRADETVVSNMSVAFDRMLLGVEDPYTFNPYHKAVREPFDYYMFVHTYIRPLIDFKNSYVGNASIFSELEDKIRQGHNIVLISNHQSEADPAVISLLLEAQSPFIGENIKCVAGDRVITDPLCKPFSMGRNLICVYSKKHMNDDPELVDMKRKANTRSLKEMATMLRSGGQLIWIAPSGGRDRPNPSTGEWFPAPFDASSVDNMRRLVEHSGAPGHIYPMSLLCYDIMPPPPQVEKEIGEKRLVGFHGTGLSIAPEINFSDVTADCESPNEAKEAYSQALYKSVNEQYEILNSAIKHRRGVEASTSRVSLSQPWN

>TcGPAT12(XP_007040713)

MSTLPLPFFSGTSTRAPFSFSLKSSPPSAASSSSSSSLVPFQRVELFRSTSTRTTRSFSCVFYSLKAKAMAELVQDKESGVAATGSSGGGRPEVEHSRTFLEARSEQELLSGIRKEVEAGRLPPNIAAGMEELYQNYRNAVFQSGDPSAVEIVLSNMAVAFDRMLLDVEDPFVFEPYHKALREPFDYYMFGQNYIRPLTDFRNSYVGNLSLFYEIEEKLKQGHNVVLISNHQTEADPAIITLLLEKTNPHVAENMIYVAGDRVITDPLCKPFSMGRNLICVYSKKHMYDVPELVEMKRKANTQSLKQMALLLRGGSKIVWIAPSGGRDRPDPLTEEWYPAPFDSSSVDNMRRLIEHSGAPGHIYPLALLCYNIMPPPPQVEKQIGERRNISFHGAGLSVAPKISFQEIAAACEKSEEAKDVYSQALYKSVTKQYNVLKSAVHGKQGLEASTAGVSLSQPWN

>TcGPAT5(XP_007009249)

MESVVSELEGVLLKDPDPFCYFMLVAFEASGLIRFALLLLFWPLIRLLDMLGLDDAGLKLMTFVATAGLREAEIESVSRAVLPKFYMDDVDMEAWKVFSSYDKRVVVTKTPRIMVARFVKEHLRADEVLGSELVVNRFGFATGFVKGDIASISSRVAKLFVDEEPTLGLGRAPSSFQFLSLCKKQMHPPLMTNQNLHDHQLLRPLPVIFHDGRLVKRPTPSTALLILLWMPLGILLATIRIVVGLILPMRVIPYMSRLFGGKIIVKGKQPPPVSGSSSGVLFVCTHRTLMDPVVLSAVLMRKIPAVTYSISRLSEILSPIPTVRLTRIREVDAEKIKRELAKGDLVVCPEGTTCREPFLLRFSALFAELTDRIVPVAMNYRVGFFHATTARGWKGLDPIFFFMNPRPVYEVTFLNQLPAEATCSSGKSPHDVANYVQRILAATLGFECTNFTRKDKYRVLAGNDGTVSCTSFVDQVKKVVSTFKPFFQ

>ath:AT4G00400 GPAT8

MSPEKKSQNFPPITECRDGEYDSIAADLDGTLLLSRSSFPYFMLVAVEAGSLLRGLILLLSLPFVIISYLFVSESLGIQILIFISFAGLKIRDIELVSRAVLPRFYAADVRKDSFEVFDKCKRKVVVTANPIVMVEAFVKDYLGGDKVLGTEIEVNPKTNRATGFVKKPGVLVGDLKRLAILKEFGNESPDLGLGDRTSDHDFMSLCKKGYMVHATKSATTIPKERLKNRIVFHDGRLAQRPTPLNAIITYLWLPFGFILSIIRVYFNLPLPERFVRYTYEMLGIHLTIRGHRPPPPSPGTLGNLYVLNHRTALDPIIVAIALGRKICCVTYSVSRLSLMLSPIPAVALTRDRATDAANMRKLLEKGDLVICPEGTTCREEYLLRFSALFAELSDRIVPVAMNCKQGMFNGTTVRGVKFWDPYFFFMNPRPSYEATFLDRLPEEMTVNGGGKTPIEVANYVQKVIGAVLGFECTELTRKDKYLLLGGNDGKVESINNTKK

>ath:AT5G06090 GPAT7

MESSTTTSYSVVSELEGTLLKNPKPFAYFMLVAFEASGLIRFATLLFLWPIIALLDVLGYRNGSLKLMIFVATAGLHESEIESVARAVLPKFFMDDISMDAWRAFGSCDKRVVVTRMPRVMVERFAKDHLSADEVIGTEIVVNRFGYATGLIQETNVDQSVFNSVANLFVDRRPQLGLGRHIISDSPTFLSLCEEQVHAPVPSNYNGHNQRLHVQPLPVIFHDGRLVKLPTPATALIILLWIPFGIILAMIRIFVGFLLPLWAIPYVSRIFNTRFIVKGKPPAQATTGNPGVLFVCTHRTLMDPVVLSYVLGRSIPAVTYSISRLSEILSPIPTFRLTRIRDVDAEMIKKELSNGDLVVYPEGTTCREPFLLRFSALFAELTDNIVPVAMNYRVGFFHATTARGWKGLDPIFFFMNPRPVYEVTFLNQLEVEATCSSGKSPYDVANYVQRILAATLGFECTNFTRKDKYRVLAGNDGTVSYLSFLDQVKKVVTTFKPFLH

>TcGPAT1(XP_007051782)

MAPAKSGRSFPSITKCEGSTYESIAADLDGTLLISRSSFPYFMLVAVEAGSLLRGLILLLSLPLVIVSYLFISEAIGIQILIFISFAGLKIRDIELVSRAVLPRFYAANVRKESFEVFDRCKRKVVVTANPTFMVEPFVKDFLGGDKVLGTEIEVNPKTKKATGFVKKPGVLVSELKRLAILKEFGEESPDLGIGDRESDHDFMSICKEGYMVHPSKSATPVPLDRLKSRIIFHDGRFVQRPDPLNALITYLWLPFGFILSIVRVYFNLPLPERIVRYTYEMLGIHLVIRGKRPPPPSPGTPGNLYVCNHRSALDPIVIAIALGRKVSCVTYSVSRLSRFLSPIPAVALTRDRAADAARISELLQKGDLVVCPEGTTCREQFLLRFSALFAELSDRIVPVAVNCRQNMFYGTTVRGVKFWDPYFFFMNPRPTYEVTFLDRLPEEMTVKAGGKSSIEVANHVQKVLGDVLGFECTGLTRKDKYLLLGGNDGKVESMYNAKK

>ath:AT2G38110 GPAT6

MGAQEKRRRFEQISKCDVKDRSNHTVAADLDGTLLISRSAFPYYFLVALEAGSLLRALILLVSVPFVYLTYLTISETLAINVFVFITFAGLKIRDVELVVRSVLPRFYAEDVRPDTWRIFNTFGKRYIITASPRIMVEPFVKTFLGVDKVLGTELEVSKSGRATGFTRKPGILVGQYKRDVVLREFGGLASDLPDLGLGDSKTDHDFMSICKEGYMVPRTKCEPLPRNKLLSPIIFHEGRLVQRPTPLVALLTFLWLPVGFVLSIIRVYTNIPLPERIARYNYKLTGIKLVVNGHPPPPPKPGQPGHLLVCNHRTVLDPVVTAVALGRKISCVTYSISKFSELISPIKAVALTRQREKDAANIKRLLEEGDLVICPEGTTCREPFLLRFSALFAELTDRIVPVAINTKQSMFNGTTTRGYKLLDPYFAFMNPRPTYEITFLKQIPAELTCKGGKSPIEVANYIQRVLGGTLGFECTNFTRKDKYAMLAGTDGRVPVKKEKT

>ath:AT3G11430 GPAT5

MVMEQAGTTSYSVVSEFEGTILKNADSFSYFMLVAFEAAGLIRFAILLFLWPVITLLDVFSYKNAALKLKIFVATVGLREPEIESVARAVLPKFYMDDVSMDTWRVFSSCKKRVVVTRMPRVMVERFAKEHLRADEVIGTELIVNRFGFVTGLIRETDVDQSALNRVANLFVGRRPQLGLGKPALTASTNFLSLCEEHIHAPIPENYNHGDQQLQLRPLPVIFHDGRLVKRPTPATALIILLWIPFGIILAVIRIFLGAVLPLWATPYVSQIFGGHIIVKGKPPQPPAAGKSGVLFVCTHRTLMDPVVLSYVLGRSIPAVTYSISRLSEILSPIPTVRLTRIRDVDAAKIKQQLSKGDLVVCPEGTTCREPFLLRFSALFAELTDRIVPVAMNYRVGFFHATTARGWKGLDPIFFFMNPRPVYEITFLNQLPMEATCSSGKSPHDVANYVQRILAATLGFECTNFTRKDKYRVLAGNDGTVSYLSLLDQLKKVVSTFEPCLH

>ath:AT1G01610 GPAT4

MSPAKKSRSFPPISECKSREYDSIAADLDGTLLLSRSSFPYFMLVAIEAGSLFRGLILLLSLPIVIIAYLFVSESLGIQILIFISFAGIKIKNIELVSRAVLTRFYAADVRKDSFEVFDKCKKRKVVVTANPIVMVEPFVKDYLGGDKVLGTEIEVNPKTMKATGFVKKPGVLVGDLKRLAILKEFGDDSPDLGLGDRTSDHDFMSICKEGYMVHETKSATTVPIESLKNRIIFHDGRLVQRPTPLNALIIYLWLPFGFMLSVFRVYFNLPLPERFVRYTYEILGIHLTIRGHRPPPPSPGKPGNLYVLNHRTALDPIIIAIALGRKITCVTYSVSRLSLMLSPIPAVALTRDRVADAARMRQLLEKGDLVICPEGTTCREPYLLRFSALFAELSDRIVPVAMNCKQGMFNGTTVRGVKFWDPYFFFMNPRPSYEATFLDRLPEEMTVNGGGKTPFEVANYVQKVIGGVLGFECTELTRKDKYLLLGGNDGKVESINKTKSME

>TcGPAT9(XP_007026769)

MVMGAHRRFEPISKCSNEGRSNQTVAADLDGTLLLSRSAFPYFMLVALEAGSLVRALVLLASVPFVYFTYLFISESVAINTFIFITFAGLKVKDIELVSRSVLPKFYAEDVHPESWRVFSSFGKRYIITASPRIMVEPFVKTFLGADKVIGTELEVTKSGRATGFTIKPGVLVGEHKRAAISKEFGMKLPDLGLGDRETDHDFMSLCKEGYMVPRSKCDPLPRNKLLSPIIFHEGRLVQRPTPVAALLTFLWLPIGFILSLLRVYLNIPLPERIARYNYKILGIKLIVKGNPPPAPKKGQSGVLFVCNHRTVLDPVVTAVALGRKISCVTYSISKFTEIISPIRAVALSREREKDAANIKRLLEEGDLVICPEGTTCREPFLLRFSALFAELTDRIVPVAINTKQTVFHGTTVRGHKLLDPYFVFMNPMPTYEITFLNQLPTELTCKGGKSAIEVANYIQRVLAGTLGFECTNLTRKDKYAMMAGTDGRVPSKKEKEQEKEKA

>TcGPAT8(XP_007032348)

MAKTKENPFPSIAQCASIGRDKHTVVADMDGTLLRGRSSFPYFALVAFEVGGILRLLFLLLASPVAGILYYFISEAAGIRVLIFATFVGMKVSDIESVARAVLPKFYSSDLHPETWRVFSSCGKSCVLTANPRVMVEAFLKDYLGADMVTGTEIHVFRGRATGLVKSPGILVGKNKVDALENAFKDKPVADIALGDRKTDYPFMKLCKESYVVPAKPKVEAVSHDKLPKPIVFHDGRLVQKPTPFMALLTILWIPVGFLLACLRIAAGALLPMPLVYYAFWALGVRVHIKGTPPPPAKKSIGQTGVLFICSHRTLLDPIFLSTALGRKIPAVTYSLSRLSEVISPIKTIRLSRDRATDASMIKKLLEAGDLVICPEGTTCREPFLLRFSALFAELTNELVPVAMANRMSMFHGTTARGWKGMDPFYFFMNPSPAYEVTFLNKLPHELTCSAGKSSHEVANYIQRMIAASLSYECTSFTRKDKYRALAGNDGTVVEKPKLSPNKVMGC

>ath:AT4G01950 GPAT3

MSAKISIFQALVFLFYRFILRRYRNSKPKYQNGPSSLLQSDLSRHTLIFNVEGALLKSDSLFPYFMLVAFEAGGVIRSFLLFILYPLISLMSHEMGVKVMVMVSFFGIKKEGFRAGRAVLPKYFLEDVGLEMFEVLKRGGKKIGVSDDLPQVMIEGFLRDYLEIDVVVGREMKVVGGYYLGIMEDKTKHDLVFDELVRKERLNTGRVIGITSFNTSLHRYLFSQFCQEIYFVKKSDKRSWQTLPRSQYPKPLIFHDGRLAIKPTLMNTLVLFMWGPFAAAAAAARLFVSLCIPYSLSIPILAFSGCRLTVTNDYVSSQKQKPSQRKGCLFVCNHRTLLDPLYVAFALRKKNIKTVTYSLSRVSEILAPIKTVRLTRDRVSDGQAMEKLLTEGDLVVCPEGTTCREPYLLRFSPLFTEVSDVIVPVAVTVHVTFFYGTTASGLKALDPLFFLLDPYPTYTIQFLDPVSGATCQDPDGKLKFEVANNVQSDIGKALDFECTSLTRKDKYLILAGNNGVVKKN

>TcGPAT13(XP_007018889)

MAKIKKLFPLKALSLLFETLLKSSGKLPYLRFKVSNGPATQFKFLKHSTLVHRTDELANQTLVFHLEGALLKSCSLFPYFMLVAFEAGGLFRALILLLLYPLVLLIGKELGLRILVFVSFVGITKEKFRAGTTILPNFFLEDVGCEGFDIVMSYKKRVAVTGMPKFMIEGFLRDYLGIDAVVARELKEFRGYFLGLVEENMDAGLVISECTHNIGLGCFRKSHDQKIFSHCKEIYLVTEAEKKNWQVLPRKRYPKPLIFHDGRLAFRPTPFAALIMFIWLPFGFLLNITRAIIFISLPFNLSTPLLALSGFIVTVSKPEPTTASINGENKPGGMLYVCNHRTLLDPLFLSGVLMNSVSAVTYSISKFSEVISPIKTVGLSRDREKDGKIMKKLLSQGDLVVCPEGTTCREPYLLRFSPLFAEMTNEIVPVAIKLQVSLFYGSTASGLKCLDSTFHLMNPNPMCSVKILNKLPSCQTHNTGGKSKFEVNNHVQNQIAAALGFECTNLTRKDKYAILAGNEGIV

>ath:AT1G02390 GPAT2

MSGNKISTLQALVFFLYRFFILRRWCHRSPKQKYQKCPSHGLHQYQDLSNHTLIFNVEGALLKSNSLFPYFMVVAFEAGGVIRSLFLLVLYPFISLMSYEMGLKTMVMLSFFGVKKESFRVGKSVLPKYFLEDVGLEMFQVLKRGGKRVAVSDLPQVMIDVFLRDYLEIEVVVGRDMKMVGGYYLGIVEDKKNLEIAFDKVVQEERLGSGRRLIGITSFNSPSHRSLFSQFCQEIYFVRNSDKKSWQTLPQDQYPKPLIFHDGRLAVKPTPLNTLVLFMWAPFAAVLAAARLVFGLNLPYSLANPFLAFSGIHLTLTVNNHNDLISADRKRGCLFVCNHRTLLDPLYISYALRKKNMKAVTYSLSRLSELLAPIKTVRLTRDRVKDGQAMEKLLSQGDLVVCPEGTTCREPYLLRFSPLFSEVCDVIVPVAIDSHVTFFYGTTASGLKAFDPIFFLLNPFPSYTVKLLDPVSGSSSSTCRGVPDNGKVNFEVANHVQHEIGNALGFECTNLTRRDKYLILAGNNGVVKKK

>TcGPAT10(XP_007031180 )

MAGKPQNLNAFLFLQKILLTKTWSKIYLQRKASNSHGFQPQFNKYPFLANGLETHSDKALMFHTEGALLKSSSVFSYFFLVAFEAGGLLRAFILFLLHPIICLVGKEWGLKIMVFVCFVGLKEESFRIGRTVLPKFFLEDVGNEGFDMVMKCGGKKIGVTDMPRVMVDCFLKDYLRVEAVVGRELKVVCGHFVGLMEEKKPSGTAFLELDELLGKENMDSSVIGISCFDNSLDQQLFSCCEVLYMVSEADKKNWQALPREKYPRPLIFHDGRLAFRPTPLASLRMFMWIPFGIFLLIVRATVGYLIPYKFSTPFLCFTGLTGTLIRPSSFPDRADDEQKQGGVLYVCNHRTLLDPIYVSIVLDKPFNAVTYSLSRMTELLAPIKTVRLTRNREEDSKMIGKLLKQGDLVICPEGTTCREPYLLRFSPLFAEITDEIVPVAIDFQVSMFYGTTASGFKFLDPFFLLMNPTSHCSASILEKLPKSLTCQGGGKSKFEVANRVQAIIAKALNFECTSLTRRDKYMILAGNQGIV

>TcGPAT4(XP_007051174)

MAKLSMEFSFFQTLFFLFCRVVFRQSKNHKSLHRNVSNIHANEGKYHKYPSFVHRSNLSNQTLVFSVEEALLKSSSLFPYFMLVAFEAGGLLRAFILFVLYPILCLVSEEMGLKIMVLVCFFGIKKKSFRVGSAVLPKFFLEDVGLEPFEMLKKGGKKVAVSKIPQVMIESFLKDYLEIDFVVGRELKEFCGYFLGVMEEKKRSKAALDEIIGSESMGFDVIGISGLKKSLDYHFFSHCKEIYQVRKADKRNWRHVPRQEYSKPLIFHDGRLALRPTLVASLTMFVWFPFGLALSILRAVVGLMLPYKISIPLLAYSGLHLFLSTPESSLHPLSLPNSKKQNPKGRLYVCNHRTLLDPVYLSFALQKDLTAVTYSLSRISELLAPIKTVRLARDRDQDGKMMEKMLNLGDLVVCPEGTTCREPYLLRFSPLFSEMSDDIVPVAMDSNVSLFYGTTASGLKCLDPLFFLMNPRPIYTVQILDGVSGLYTCHDGQRSRFKVANQVQNEIGKALGFECTKLTRRDKYLIMAGNEGIISQT

>TcGPAT3(XP_007048860)

MVFPVVFLKLADWVLYQLLANSCYRAARKMRNYGFFLRNQTLRSPPQQQAASLFPSVTKCDVGNSRRFDTLVCDIHGVLLGSDTFFPYFMLVAFEGGSIVRAFLLLLSCSFLWVLDSELKLRIMIFISFCGLRKKDIESVGRAVLPKFYLENLNLQVYEVWSKTSSRVVFTSIPRVMVEGFLHEYMSASGVVGTELHTVGNRFTGLLSSSGLLVKHNALKEHFGDKKPDVGLGSSSLHDQYFISLCKEAYVVNMEDGKSNLSSFMPRDKYPKPLIFHDGRLAFLPTPFATLSMFLWLPFGIVLSILRIFVGICLPYKLAVICATLSGVQLKFQGCFPSSNSQHKKGVLYVCTHRTLLDPVFLSTALCKPLTAVTYSLSKMSELIAPIKTVRLTRDRKQDGETMQKLLSEGDLVVCPEGTTCREPYLLRFSSLFAELADEIVPVAINAHVSMFYGTTASGLKCLDPIFFLMNPRPSYHVQILGKVPQEFTCAGGRSSLEVANYIQRKLADALGFECTTLTRRDKYLMLAGNEGVVHENKRN

>TcGPAT11(XP_007018890)

MANRQSVLWGLKTVCLVHQHLNSTIYGNLCQQAIKVIKNDDVASGINEPLSILNQRKLPYLRFKVSNGPATQFKFLKHSTLVHRTDELANQTLAFHLEGALLKSCSLFPYFMLMAFEAGGLFRALILLLLYPLVLLIGKELGLRILVFVSFVGIRKEKFRAGTAILPKFFLEDVGCEGFDIVMSYKKRVAVTGMPKFMVEGFLRDYLGIDAVVARELKEFRGYFLGLMEEKMDAGLVISECTHHIGLGCFRKSHDQKIFSHCKEIYLVTEAEKKNWQVLPRKRYPKPLIFHDGRLAFRPTPLAALIMFIWLPFGFLLNITRTIVFISLPFKLSIPLLALSGCIITVSKPEPTTASINGENKPGGMLYVCNHRTLLDPLFLSAVLMKSVCAVTYSISRFSEVISPIKTVRLSRDREKDGKIMKKLLSQGDLVVCPEGTTCREPYLLRFSPLFAEMTDEIVPVAIKLQVSLFYGSTASGLKCLDSTFHLMNPNPMCSVKILNKLPSCQTHNTGGKSKFEVTNHVQNQIAAALGFECTNLTRKDKYAILAGNEGIV

>ath:AT1G06520 GPAT1

MVLPELLVILAEWVLYRLLAKSCYRAARKLRGYGFQLKNLLSLSKTQSLHNNSQHHLHNHHQQNHPNQTLQDSLDPLFPSLTKYQELLLDKNRACSVSSDHYRDTFFCDIDGVLLRQHSSKHFHTFFPYFMLVAFEGGSIIRAILLLLSCSFLWTLQQETKLRVLSFITFSGLRVKDMDNVSRSVLPKFFLENLNIQVYDIWARTEYSKVVFTSLPQVLVERFLREHLNADDVIGTKLQEIKVMGRKFYTGLASGSGFVLKHKSAEDYFFDSKKKPALGIGSSSSPQDHIFISICKEAYFWNEEESMSKNNALPRERYPKPLIFHDGRLAFLPTPLATLAMFIWLPIGFLLAVFRISVGVFLPYHVANFLASMSGVRITFKTHNLNNGRPEKGNSGVLYVCNHRTLLDPVFLTTSLGKPLTAVTYSLSKFSEFIAPLKTVSLKRDRKKDGEAMQRLLSKGDLVVCPEGTTCREPYLLRFSPLFAELTEDIVPVAVDARVSMFYGTTASGLKCLDPIFFLMNPRPVYCLEILKKLPKEMTCAGGKSSFEVANFIQGELARVLGFECTNLTRRDKYLVLAGNEGIVR

>TcGPAT6(XP_007044411)

MKLSRRQTVLTNDNNNCNLKLAAWPCSFGYKRRKARQTSAIQPKYQVPTASYSSATAQRSKASPRLIHWLAVKDSWKSRALNVLILLFLNMAINAFPTVQNCASIGREKHTMVADMDGTLLISRSSFPYFALVAFEVGGVLRLFFLLLASPLAGLLYYFVSESAGIQVLIFATYVGMKVSDIESVARAVLPKFYSSDLHPESWRVFSSCGKRCVLTANPRIMVEPFLKDFLGADMVLGTEIGIYRGRATGFVCQPGVLVGKNKADALQKAFGEALPDVGLGDRHTDIPFIALCKEGYIVPPKPEAKAVRSEKLPKPVIFHDGRLVQKPTPLMALLIIIWIPIGFLLACLRITAGSLLPMPLVYYAFWALGVRVTVKGTPPPPVKKSTGQSGILFICSHRTLLDPIFLSTALGRPIPVVTYSVSRLSEIISPIKTVSLSRDRATDASMIKKLLQEGDLAICPEGTTCREPFLLRFSALFAELTDQHVPVAMVNRMSMFHGTTARGWKGMDPFYFFMNPSPAYEVTFLNKLPMELTCSSGKSSHEIANYIQRVIAATLSYECTSFTRKDKYRALAGNDGTVVEKPKFSANKVMGC

>GPT2(NP_012993)

MSAPAADHNAAKPIPHVPQASRRYKNSYNGFVYNIHTWLYDVSVFLFNILFTIFFREIKVRGAYNVPEVGVPTILVCAPHANQFIDPALVMSQTRLLKTSAGKSRSRMPCFVTAESSFKKRFISFFGHAMGGIPVPRIQDNLKPVDENLEIYAPDLKNHPEIIKGRSKNPQTTPVNFTKRFSAKSLLGLPDYLSNAQIKEIPDDETIILSSPFRTSKSKVVELLTNGTNFKYAEKIDNTETFQSVFDHLHTKGCVGIFPEGGSHDRPSLLPIKAGVAIMALGAVAADPTMKVAVVPCGLHYFHRNKFRSRAVLEYGEPIVVDGKYGEMYKDSPRETVSKLLKKITNSLFSVTENAPDYDTLMVIQAARRLYQPVKVRLPLPAIVEINRRLLFGYSKFKDDPRIIHLKKLVYDYNRKLDSVGLKDHQVMQLKTTKLEALRCFVTLIVRLIKFSVFAILSLPGSILFTPIFIICRVYSEKKAKEGLKKSLVKIKGTDLLATWKLIVALILAPILYVTYSILLIILARKQHYCRIWVPSNNAFIQFVYFYALLVFTTYSSLKTGEIGVDLFKSLRPLFVSIVYPGKKIEEIQTTRKNLSLELTAVCNDLGPLVFPDYDKLATEIFSKRDGYDVSSDAESSISRMSVQSRSRSSSIHSIGSLASNALSRVNSRGSLTDIPIFSDAKQGQWKSEGETSEDEDEFDEKNPAIVQTARSSDLNKENSRNTNISSKIASLVRQKREHEKKE

>SCT1(NP_009542)

MPAPKLTEKFASSKSTQKTTNYSSIEAKSVKTSADQAYIYQEPSATKKILYSIATWLLYNIFHCFFREIRGRGSFKVPQQGPVIFVAAPHANQFVDPVILMGEVKKSVNRRVSFLIAESSLKQPPIGFLASFFMAIGVVRPQDNLKPAEGTIRVDPTDYKRVIGHDTHFLTDCMPKGLIGLPKSMGFGEIQSIESDTSLTLRKEFKMAKPEIKTALLTGTTYKYAAKVDQSCVYHRVFEHLAHNNCIGIFPEGGSHDRTNLLPLKAGVAIMALGCMDKHPDVNVKIVPCGMNYFHPHKFRSRAVVEFGDPIEIPKELVAKYHNPETNRDAVKELLDTISKGLQSVTVTCSDYETLMVVQTIRRLYMTQFSTKLPLPLIVEMNRRMVKGYEFYRNDPKIADLTKDIMAYNAALRHYNLPDHLVEEAKVNFAKNLGLVFFRSIGLCILFSLAMPGIIMFSPVFILAKRISQEKARTALSKSTVKIKANDVIATWKILIGMGFAPLLYIFWSVLITYYLRHKPWNKIYVFSGSYISCVIVTYSALIVGDIGMDGFKSLRPLVLSLTSPKGLQKLQKDRRNLAERIIEVVNNFGSELFPDFDSAALREEFDVIDEEEEDRKTSELNRRKMLRKQKIKRQEKDSSSPIISQRDNHDAYEHHNQDSDGVSLVNSDNSLSNIPLFSSTFHRKSESSLASTSVAPSSSSEFEVENEILEEKNGLASKIAQAVLNKRIGENTAREEEEEEEEEEEEEEEEEEGKEGDA

>hsa:150763 GPAT2

MATMLEGRCQTQPRSSPSGREASLWSSGFGMKLEAVTPFLGKYRPFVGRCCQTCTPKSWESLFHRSITDLGFCNVILVKEENTRFRGWLVRRLCYFLWSLEQHIPPCQDVPQKIMESTGVQNLLSGRVPGGTGEGQVPDLVKKEVQRILGHIQAPPRPFLVRLFSWALLRFLNCLFLNVQLHKGQMKMVQKAAQAGLPLVLLSTHKTLLDGILLPFMLLSQGLGVLRVAWDSRACSPALRALLRKLGGLFLPPEASLSLDSSEGLLARAVVQAVIEQLLVSGQPLLIFLEEPPGALGPRLSALGQAWVGFVVQAVQVGIVPDALLVPVAVTYDLVPDAPCDIDHASAPLGLWTGALAVLRSLWSRWGCSHRICSRVHLAQPFSLQEYIVSARSCWGGRQTLEQLLQPIVLGQCTAVPDTEKEQEWTPITGPLLALKEEDQLLVRRLSCHVLSASVGSSAVMSTAIMATLLLFKHQKLLGEFSWLTEEILLRGFDVGFSGQLRSLLQHSLSLLRAHVALLRIRQGDLLVVPQPGPGLTHLAQLSAELLPVFLSEAVGACAVRGLLAGRVPPQGPWELQGILLLSQNELYRQILLLMHLLPQDLLLLKPCQSSYCYCQEVLDRLIQCGLLVAEETPGSRPACDTGRQRLSRKLLWKPSGDFTDSDSDDFGEADGRYFRLSQQSHCPDFFLFLCRLLSPLLKAFAQAAAFLRQGQLPDTELGYTEQLFQFLQATAQEEGIFECADPKLAISAVWTFRDLGVLQQTPSPAGPRLHLSPTFASLDNQEKLEQFIRQFICS

>hsa:57678

MDESALTLGTIDVSYLPHSSEYSVGRCKHTSEEWGECGFRPTIFRSATLKWKESLMSRKRPFVGRCCYSCTPQSWDKFFNPSIPSLGLRNVIYINETHTRHRGWLARRLSYVLFIQERDVHKGMFATNVTENVLNSSRVQEAIAEVAAELNPDGSAQQQSKAVNKVKKKAKRILQEMVATVSPAMIRLTGWVLLKLFNSFFWNIQIHKGQLEMVKAATETNLPLLFLPVHRSHIDYLLLTFILFCHNIKAPYIASGNNLNIPIFSTLIHKLGGFFIRRRLDETPDGRKDVLYRALLHGHIVELLRQQQFLEIFLEGTRSRSGKTSCARAGLLSVVVDTLSTNVIPDILIIPVGISYDRIIEGHYNGEQLGKPKKNESLWSVARGVIRMLRKNYGCVRVDFAQPFSLKEYLESQSQKPVSALLSLEQALLPAILPSRPSDAADEGRDTSINESRNATDESLRRRLIANLAEHILFTASKSCAIMSTHIVACLLLYRHRQGIDLSTLVEDFFVMKEEVLARDFDLGFSGNSEDVVMHAIQLLGNCVTITHTSRNDEFFITPSTTVPSVFELNFYSNGVLHVFIMEAIIACSLYAVLNKRGLGGPTSTPPNLISQEQLVRKAASLCYLLSNEGTISLPCQTFYQVCHETVGKFIQYGILTVAEHDDQEDISPSLAEQQWDKKLPEPLSWRSDEEDEDSDFGEEQRDCYLKVSQSKEHQQFITFLQRLLGPLLEAYSSAAIFVHNFSGPVPEPEYLQKLHKYLITRTERNVAVYAESATYCLVKNAVKMFKDIGVFKETKQKRVSVLELSSTFLPQCNRQKLLEYILSFVVL

**LPAT genes used for phylogenetic tree construction of Fig 3b.**

>TcLPAT8 (XP_007020859 )

MWILGNPIKIEGTEFSDERAIYISNHASPIDIFLIMWLTPTGTVGIAKKEIIWYPLFGQLYVLANHLRIDRSSPSTAIQSMKEAIQAVIKHNLSLIIFPEGTRSKNGRLLPFKKGFVHLALQSHLPIVPIVLAGTHLAWRKGSLHVRPAPISVKYLPPISTGSWKDDKIDDYIKMVHDIYVESLPEPQKPIVSEDTTNSS

>hsa:10555

MELWPCLAAALLLLLLLVQLSRAAEFYAKVALYCALCFTVSAVASLVCLLRHGGRTVENMSIIGWFVRSFKYFYGLRFEVRDPRRLQEARPCVIVSNHQSILDMMGLMEVLPERCVQIAKRELLFLGPVGLIMYLGGVFFINRQRSSTAMTVMADLGERMVRENVPIVPVVYSSFSSFYNTKKKFFTSGTVTVQVLEAIPTSGLTAADVPALVDTCHRAMRTTFLHISKTPQENGATAGSGVQPAQ

>hsa:10554

MDLWPGAWMLLLLLFLLLLFLLPTLWFCSPSAKYFFKMAFYNGWILFLAVLAIPVCAVRGRNVENMKILRLMLLHIKYLYGIRVEVRGAHHFPPSQPYVVVSNHQSSLDLLGMMEVLPGRCVPIAKRELLWAGSAGLACWLAGVIFIDRKRTGDAISVMSEVAQTLLTQDVRVWVFPEGTRNHNGSMLPFKRGAFHLAVQAQVPIVPIVMSSYQDFYCKKERRFTSGQCQVRVLPPVPTEGLTPDDVPALADRVRHSMLTVFREISTDGRGGGDYLKKPGGGG

>SLC1 (NP_010231)

MSVIGRFLYYLRSVLVVLALAGCGFYGVIASILCTLIGKQHLAQWITARCFYHVMKLMLGLDVKVVGEENLAKKPYIMIANHQSTLDIFMLGRIFPPGCTVTAKKSLKYVPFLGWFMALSGTYFLDRSKRQEAIDTLNKGLENVKKNKRALWVFPEGTRSYTSELTMLPFKKGAFHLAQQGKIPIVPVVVSNTSTLVSPKYGVFNRGCMIVRILKPISTENLTKDKIGEFAEKVRDQMVDTLKEIGYSPAINDTTLPPQAIEYAALQHDKKVNKKIKNEPVPSVSISNDVNTHNEGSSVKKMH

>TcLPAT2 (XP_007020857)

MESSGSGSFLRNRRLGSFLDTNSDPNVRETQKVSSKGGARQSPKTDDAFVDDDGWICSLISCVRIVACFLTMMVTTFIWALIMLLLLPWPSQRIRQGNIYGHVTGRLLMWILGNPIKIEGTEFSNERAIYICNHASPIDIFLIMWLTPTGTVGIAKKEIIWYPLFGQLYVLANHLRIDRSNPSTAIQSMKEAVQAVIKHNLSLIIFPEGTRSKNGRLLPFKKGFVHLALQSHIPIVPIVLTGTHLAWRKGSLHVRPAPISVKYLPPISADSWKDDKIDDYIKMVHDIYVENLPEPQKPIVSEDTTNSSRS

>hsa:51099

MAAEEEEVDSADTGERSGWLTGWLPTWCPTSISHLKEAEEKMLKCVPCTYKKEPVRISNGNKIWTLKFSHNISNKTPLVLLHGFGGGLGLWALNFGDLCTNRPVYAFDLLGFGRSSRPRFDSDAEEVENQFVESIEEWRCALGLDKMILLGHNLGGFLAAAYSLKYPSRVNHLILVEPWGFPERPDLADQDRPIPVWIRALGAALTPFNPLAGLRIAGPFGLSLVQRLRPDFKRKYSSMFEDDTVTEYIYHCNVQTPSGETAFKNMTIPYGWAKRPMLQRIGKMHPDIPVSVIFGARSCIDGNSGTSIQSLRPHSYVKTIAILGAGHYVYADQPEEFNQKVKEICDTVD

>ath:AT4G30580 ATS2

MDVASARSISSHPSYYGKPICSSQSSLIRISRDKVCCFGRISNGMTSFTTSLHAVPSEKFMGETRRTGIQWSNRSLRHDPYRFLDKKSPRSSQLARDITVRADLSGAATPDSSFPEPEIKLSSRLRGIFFCVVAGISATFLIVLMIIGHPFVLLFDPYRRKFHHFIAKLWASISIYPFYKINIEGLENLPSSDTPAVYVSNHQSFLDIYTLLSLGKSFKFISKTGIFVIPIIGWAMSMMGVVPLKRMDPRSQVDCLKRCMELLKKGASVFFFPEGTRSKDGRLGSFKKGAFTVAAKTGVAVVPITLMGTGKIMPTGSEGILNHGNVRVIIHKPIHGSKADVLCNEARSKIAESMDL

>TcLPAT1 (XP_007011912)

MELSSLPSVSSLSLCHPKPRSSATLPFLPFSNRKGAYFGYSLCKRATLRNSCNSAQNNFLRISRTHDGVSRCYFNQKEGLNRSYYNSKLHNENKLSRYIVARSEFAGTGTSDAAYSLSEIKPGSKVRGVCFYAVTAIAAILLIWFMLVLHPFVLLFDRYRRKAQHFIAKLWAMATVAPFFKIEFEGLENLPPQDVPAVYVSNHQSFLDIYTLLTLGRSFKFISKTGIFLYPIIGWAMSMMGLIPLKRMDSRSQLDCLKRCMDLIRNGASVFFFPEGTRSKDGKLGSFKKGAFSVAAKTGVPVVPMTLIGTGKIMPLGLEGVINSGSVKVVIHKPIKGSDPEILCNEARNTIADTLKHQC

>TcLPAT5(XP_007015027)

MEISAIVTSVPLSLMFIFIGTIINLIQVACYLTIRPLSKSTFRRINGAVSEVLWLELVWLMEWWSGYLKTYRLMGKEHALLMPNHVADADTMLVWLLAQRMGCLRSALIISKKSTQYLPIFGWTTWFYEFIFVDRNWAKDGSKLKSSFQALKDFPIPFWVTIFAEGTRLTPDKLLEAQTFASTKGLAIPKNVLIPRTKGFVTAVQSLRSFVPALYDVTIAIQKGHPSPSLLRFLKRQPCKIKVHIKRYSMKELPESDEGIAQWCRNRFIAKDALLDKFAATGRFDEEEITDFRRSTKSLIVFLITLFLFSVGAWISCQKFSLLSNRRGYTILATIVGTAAIFAHIFLEFTKLPPQKCKATLQTNGDT

>TcLPAT4 (XP_007042726 )

MEVPSANHEMRHRSLTPLRVFRGLICLLVLFSTAFMMIVYCGFLTTVIFRLFSIHYSRKATSFFFSAWLSLWPFLFEKINKTKVIFSGDDVPPRERVLLICNHRTEVDWMYLWDFALRKGCLGYIKYILKSSLMKLPVFGWAFHILEFIPVERKWEVDESNMRNMLSTFKDPQDPLWLVLFPEGTDFTEQKCLRSQKYAAENGLPILKNLLLPKSKGFFACLEDLRSSLDAVYDVTIGYKHCCPSFLDNVFGVDPSEVHIHIRRITLDDIPISERELTAWLMDTFQHKDQLLSNFKSEGYFPRQGPEVNLSAVKCIVDVVLVLFLTSAFIFFTFFSSIWFKIFVSLSCAYMTSATYLNTRPVPVFSLVKTCV

>ath:AT3G18850 LPAT5

MEKKSVPNSDKLSLIRVLRGIICLMVLVSTAFMMLIFWGFLSAVVLRLFSIRYSRKCVSFFFGSWLALWPFLFEKINKTKVIFSGDKVPCEDRVLLIANHRTEVDWMYFWDLALRKGQIGNIKYVLKSSLMKLPLFGWAFHLFEFIPVERRWEVDEANLRQIVSSFKDPRDALWLALFPEGTDYTEAKCQRSKKFAAENGLPILNNVLLPRTKGFVSCLQELSCSLDAVYDVTIGYKTRCPSFLDNVYGIEPSEVHIHIRRINLTQIPNQEKDINAWLMNTFQLKDQLLNDFYSNGHFPNEGTEKEFNTKKYLINCLAVIAFTTICTHLTFFSSMIWFRIYVSLACVYLTSATHFNLRSVPLVETAKNSLKLVNK

>ath:AT1G51260 LPAT3

MKIPAALVFIPVGVLFLISGLIVNIIQLVFFIIVRPFSRSLYRRINKNVAELLWLQLIWLFDWWACIKINLYVDAETLELIGKEHALVLSNHRSDIDWLIGWVMAQRVGCLGSSLAIMKKEAKYLPIIGWSMWFSDYIFLERSWAKDENTLKAGFKRLEDFPMTFWLALFVEGTRFTQEKLEAAQEYASIRSLPSPRNVLIPRTKGFVSAVSEIRSFVPAIYDCTLTVHNNQPTPTLLRMFSGQSSEINLQMRRHKMSELPETDDGIAQWCQDLFITKDAQLEKYFTKDVFSDLEVHQINRPIKPLIVVIIWLGFLVFGGFKLLQWLSIVASWKIILLFVFFLVIATITMQILIQSSESQRSTPAKRPLQEQLISA

>hsa:56894

MGLLAFLKTQFVLHLLVGFVFVVSGLVINFVQLCTLALWPVSKQLYRRLNCRLAYSLWSQLVMLLEWWSCTECTLFTDQATVERFGKEHAVIILNHNFEIDFLCGWTMCERFGVLGSSKVLAKKELLYVPLIGWTWYFLEIVFCKRKWEEDRDTVVEGLRRLSDYPEYMWFLLYCEGTRFTETKHRVSMEVAAAKGLPVLKYHLLPRTKGFTTAVKCLRGTVAAVYDVTLNFRGNKNPSLLGILYGKKYEADMCVRRFPLEDIPLDEKEAAQWLHKLYQEKDALQEIYNQKGMFPGEQFKPARRPWTLLNFLSWATILLSPLFSFVLGVFASGSPLLILTFLGFVGAASFGVRRLIGVTEIEKGSSYGNQEFKKKE

>hsa:253558

MVSWKGIYFILTLFWGSFFGSIFMLSPFLPLMFVNPSWYRWINNRLVATWLTLPVALLETMFGVKVIITGDAFVPGERSVIIMNHRTRMDWMFLWNCLMRYSYLRLEKICLKASLKGVPGFGWAMQAAAYIFIHRKWKDDKSHFEDMIDYFCDIHEPLQLLIFPEGTDLTENSKSRSNAFAEKNGLQKYEYVLHPRTTGFTFVVDRLREGKNLDAVHDITVAYPHNIPQSEKHLLQGDFPREIHFHVHRYPIDTLPTSKEDLQLWCHKRWEEKEERLRSFYQGEKNFYFTGQSVIPPCKSELRVLVVKLLSILYWTLFSPAMCLLIYLYSLVKWYFIITIVIFVLQERIFGGLEIIELACYRLLHKQPHLNSKKNE

>ath:AT1G75020 LPAT4

MEVCGDLKSDNLKNRPLTPLRILRGLMILLVFLSTAFMFLLYFAPIAALGLRLLSVQQSRKVVSLIFGLWLALWPYLFETVNGTTVVFSGDIIPVEKRVLLIANHRTEVDWMYLWNIALRKGCLGYIKYVLKSSLMKLPIFGWGFHVLEFIPVERKREVDEPVLLQMLSSFKDPQEPLWLALFPEGTDFTEEKCKRSQKFAAEVGLPALSNVLLPKTRGFGVCLEVLHNSLDAVYDLTIAYKPRCPSFMDNVFGTDPSEVHIHVRRVLLKEIPANEAESSAWLMDSFKLKDKLLSDFNAQGKFPNQRPEEELSVLKCIATFAGVISLTVVFIYLTLYSHSCFKVYACLSGTYLTFATYYKFQPSPGCFREDSCKVKNH

>hsa:56895

MDLAGLLKSQFLCHLVFCYVFIASGLIINTIQLFTLLLWPINKQLFRKINCRLSYCISSQLVMLLEWWSGTECTIFTDPRAYLKYGKENAIVVLNHKFEIDFLCGWSLSERFGLLGGSKVLAKKELAYVPIIGWMWYFTEMVFCSRKWEQDRKTVATSLQHLRDYPEKYFFLIHCEGTRFTEKKHEISMQVARAKGLPRLKHHLLPRTKGFAITVRSLRNVVSAVYDCTLNFRNNENPTLLGVLNGKKYHADLYVRRIPLEDIPEDDDECSAWLHKLYQEKDAFQEEYYRTGTFPETPMVPPRRPWTLVNWLFWASLVLYPFFQFLVSMIRSGSSLTLASFILVFFVASVGVRWMIGVTEIDKGSAYGNSDSKQKLND

>TcLPAT3 (XP_007017453 )

MEVCRPLKPDDKLKHRPLTPFRFLRGLICLVVFLLTAFMFLAYLGPGAVLLRFFSLHYCRKATSFFFGLWLALWPFLFEKINRTKVVFSGDNAPQKERVLLIVNHRTEVDWMYLWDLAMRKGCLGYIKYILKSSLMKLPVLGWGFHILEFISVDRKWETDENVLRQMLSTFKNPRDPLWLALFPEGTDFTEEKCRNSQKFAAEVGLPVLTNVLLPRTRGFCLCLETLRDSLDAVYDLSIAYKHQCPFFLDNVFGVDPSEVHIHVRRIPVKEIPTSNAEAAAWLIDTFKLKDQLLSDFKSQGHFPNQGTQQELSSLKSLLNLTVIISLTAIFTYLTFSSNLYMIYVSLACLYLAYITHYKIRPMPVLSSVKPLSYPKGKRDE

>TcLPAT7 (XP_007015031)

MAIPAALVVVPVGVLFILSGLIVNLIQIYFQAVLLVLLRPLSKSLYRRTNKVIVELLWLELIWLIDWWASIKIDLYTDAATFQLMGKEHALVICNHRSDIDWLVGWVLAQRSSCLGSALAIIKRSAKFLPIIGWSMWFSDYVFLERSWAKDKETLKFGFKQLEDFPMPFWLALFVEGTRFTQAKLLLAQDYATSKGLPIPRNVLIPRTKGFVSAVYHMRSFVPVIYDCTVAIPKNQPPPTMLRMFRGQPSVVNLQIRRHLMQELPETIDGIAQWCKDIFVTKDAMLEKYFTTDAFSDLEYQDIGRPKKSLIVVISWSCLLLLGIIKLFQWFSFLASWEVIAFSVTFLVIVTIVMQILIHSSESKRSTPPKVFQVDSIKEKLLC

>ath:AT3G57650 LPAT2

MVIAAAVIVPLGLLFFISGLAVNLFQAVCYVLIRPLSKNTYRKINRVVAETLWLELVWIVDWWAGVKIQVFADNETFNRMGKEHALVVCNHRSDIDWLVGWILAQRSGCLGSALAVMKKSSKFLPVIGWSMWFSEYLFLERNWAKDESTLKSGLQRLSDFPRPFWLALFVEGTRFTEAKLKAAQEYAASSELPIPRNVLIPRTKGFVSAVSNMRSFVPAIYDMTVTIPKTSPPPTMLRLFKGQPSVVHVHIKCHSMKDLPESDDAIAQWCRDQFVAKDALLDKHIAADTFPGQQEQNIGRPIKSLAVVLSWACVLTLGAIKFLHWAQLFSSWKGITISALGLGIITLCMQILIRSSQSERSTPAKVVPAKPKDNHHPESSSQTETEKEK

>TcLPAT10 (XP_007023106)

MELDMGTMAASIGVSVPVLRFLLCFVATILSKNTYRKINRVVAELLWLELVWLVDWWAGVKIKVFMDPESFNLMGKEHALVVANHRSDIDWLVGWLLAQRSGCLGSALAVMKKSSKFLPVYVVVICLKFQLSIVIGWSMWFSEYLFLERSWAKDENTLKAGLQRLKDFPRPFWLAFFVEGTRFTQAKFLAAQEYAASQGLPIPRNVLIPRTKGFVSAVSHMRSFVPAIYDMTVAIPKSSPSPTMLRLFKGQPSVVHVHIKRCLMKELPETDEAVAQWCKDMFVEKDKLLDKHIAEDTFSDQPMQDLGRPIKSLLVVASWACLMAYGALKFLQCSSLLSSWKGIAFFLVGLAIVTILMHILILFSQSERSTPAKVAPGKPKNDGETSEARRDKQQ

>TcLPAT6 (XP_007028850.1)

MAIAAAAVIVPLGLLFFISGLVVNLIQALCFVLIRPLSKNTYRKINRVVAELLWLELVWLVDWWAGVKIKVFMDPESFNLMGKEHALVVANHRSDIDWLVGWLLAQRSGCLGSALAVMKKSSKFLPVYVVVICLKFQLSIVIGWSMWFSEYLFLERSWAKDENTLKAGLQRLKDFPRPFWLAFFVEGTRFTQAKFLAAQEYAASQGLPIPRNVLIPRTKGFVSAVSHMRSFVPAIYDMTVAIPKSSPSPTMLRLFKGQPSVVHVHIKRCLMKELPETDEAVAQWCKDMFVEKDKLLDKHIAEDTFSDQPMQDLGRPIKSLLVVASWACLMAYGALKFLQCSSLLSSWKGIAFFLVGLAIVTILMHILILFSQSERSTPAKVAPGKPKNDGETSEARRDKQQ

>ath:AT1G12640

MDMSSMAGSIGVSVAVLRFLLCFVATIPVSFACRIVPSRLGKHLYAAASGAFLSYLSFGFSSNLHFLVPMTIGYASMAIYRPKCGIITFFLGFAYLIGCHVFYMSGDAWKEGGIDSTGALMVLTLKVISCSMNYNDGMLKEEGLREAQKKNRLIQMPSLIEYFGYCLCCGSHFAGPVYEMKDYLEWTEGKGIWDTTEKRKKPSPYGATIRAILQAAICMALYLYLVPQYPLTRFTEPVYQEWGFLRKFSYQYMAGFTARWKYYFIWSISEASIIISGLGFSGWTDDASPKPKWDRAKNVDILGVELAKSAVQIPLVWNIQVSTWLRHYVYERLVQNGKKAGFFQLLATQTVSAVWHGLYPGYMMFFVQSALMIAGSRVIYRWQQAISPKMAMLRNIMVFINFLYTVLVLNYSAVGFMVLSLHETLTAYGSVYYIGTIIPVGLILLSYVVPAKPSRPKPRKEE

>ath:AT1G63050

MELLDMNSMAASIGVSVAVLRFLLCFVATIPISFLWRFIPSRLGKHIYSAASGAFLSYLSFGFSSNLHFLVPMTIGYASMAIYRPLSGFITFFLGFAYLIGCHVFYMSGDAWKEGGIDSTGALMVLTLKVISCSINYNDGMLKEEGLREAQKKNRLIQMPSLIEYFGYCLCCGSHFAGPVFEMKDYLEWTEEKGIWAVSEKGKRPSPYGAMIRAVFQAAICMALYLYLVPQFPLTRFTEPVYQEWGFLKRFGYQYMAGFTARWKYYFIWSISEASIIISGLGFSGWTDETQTKAKWDRAKNVDILGVELAKSAVQIPLFWNIQVSTWLRHYVYERIVKPGKKAGFFQLLATQTVSAVWHGLYPGYIIFFVQSALMIDGSKAIYRWQQAIPPKMAMLRNVLVLINFLYTVVVLNYSSVGFMVLSLHETLVAFKSVYYIGTVIPIAVLLLSYLVPVKPVRPKTRKEE

>hsa:154141

MAAEPQPSSLSYRTTGSTYLHPLSELLGIPLDQVNFVVCQLVALFAAFWFRIYLRPGTTSSDVRHAVATIFGIYFVIFCFGWYSVHLFVLVLMCYAIMVTASVSNIHRYSFFVAMGYLTICHISRIYIFHYGILTTDFSGPLMIVTQKITTLAFQVHDGLGRRAEDLSAEQHRLAIKVKPSFLEYLSYLLNFMSVIAGPCNNFKDYIAFIEGKHIHMKLLEVNWKRKGFHSLPEPSPTGAVIHKLGITLVSLLLFLTLTKTFPVTCLVDDWFVHKASFPARLCYLYVVMQASKPKYYFAWTLADAVNNAAGFGFSGVDKNGNFCWDLLSNLNIWKIETATSFKMYLENWNIQTATWLKCVCYQRVPWYPTVLTFILSALWHGVYPGYYFTFLTGILVTLAARAVRNNYRHYFLSSRALKAVYDAGTWAVTQLAVSYTVAPFVMLAVEPTISLYKSMYFYLHIISLLIILFLPMKPQAHTQRRPQTLNSINKRKTD

>hsa:129642 MBOAT2, LPCAT4, OACT2

MATTSTTGSTLLQPLSNAVQLPIDQVNFVVCQLFALLAAIWFRTYLHSSKTSSFIRHVVATLLGLYLALFCFGWYALHFLVQSGISYCIMIIIGVENMHNYCFVFALGYLTVCQVTRVYIFDYGQYSADFSGPMMIITQKITSLACEIHDGMFRKDEELTSSQRDLAVRRMPSLLEYLSYNCNFMGILAGPLCSYKDYITFIEGRSYHITQSGENGKEETQYERTEPSPNTAVVQKLLVCGLSLLFHLTICTTLPVEYNIDEHFQATASWPTKIIYLYISLLAARPKYYFAWTLADAINNAAGFGFRGYDENGAARWDLISNLRIQQIEMSTSFKMFLDNWNIQTALWLKRVCYERTSFSPTIQTFILSAIWHGVYPGYYLTFLTGVLMTLAARAMRNNFRHYFIEPSQLKLFYDVITWIVTQVAISYTVVPFVLLSIKPSLTFYSSWYYCLHILGILVLLLLPVKKTQRRKNTHENIQLSQSKKFDEGENSLGQNSFSTTNNVCNQNQEIASRHSSLKQ

>SLC4 (NP_014818)

MYNPVDAVLTKIITNYGIDSFTLRYAICLLGSFPLNAILKRIPEKRIGLKCCFIISMSMFYLFGVLNLVSGFRTLFISTMFTYLISRFYRSKFMPHLNFMFVMGHLAINHIHAQFLNEQTQTTVDITSSQMVLAMKLTSFAWSYYDGSCTSESDFKDLTEHQKSRAVRGHPPLLKFLAYAFFYSTLLTGPSFDYADFDSWLNCEMFRDLPESKKPMRRHHPGERRQIPKNGKLALWKVVQGLAWMILSTLGMKHFPVKYVLDKDGFPTRSFIFRIHYLFLLGFIHRFKYYAAWTISEGSCILCGLGYNGYDSKTQKIRWDRVRNIDIWTVETAQNTREMLEAWNMNTNKWLKYSVYLRVTKKGKKPGFRSTLFTFLTSAFWHGTRPGYYLTFATGALYQTCGKIYRRNFRPIFLREDGVTPLPSKKIYDLVGIYAIKLAFGYMVQPFIILDLKPSLMVWGSVYFYVHIIVAFSFFLFRGPYAKQVTEFFKSKQPKEIFIRKQKKLEKDISASSPNLGGILKAKIEHEKGKTAEEEEMNLGIPPIELEKWDNAKEDWEDFCKDYKEWRNKNGLEIEEENLSKAFERFKQEFSNAASGSGERVRKMSFSGYSPKPISKKEE

>ath:AT3G57140 SDP1LIKE

MDISNEAGVDAFSIIGPTTIIGRTIAVRILFCNSVSIFRHKVFRILKFFLRGGRVLLSPFVSLLHPRNPQGILVMVTTMAFLLNRYTSLKAKAEMAYRRKFWRNMMRAALTYEEWSHAAKMLDKETPKVNETDLFDVELVSNKLDELKHRRHEGSLRDIIFCMRADLVRNLGNMCNPELHKGRLHVPRLIKEYIDEVSTQLRMVCDMDTEELSLEEKLSFMHETRHAYGRTALLLSGGASLGAFHLGVVKTLVEHKLLPRIIAGSSVGSVMCAVVGTRSWPELQSFFEGSWHALQFFDQMGGIFTTVKRVMTQGAVHEIRHLQWKLRNLTNNLTFQEAYDITGRILGITVCSLRKHEPPRCLNYLTSPHVVIWSAVTASCAFPGLFEAQELMAKDRTGEIVPYHPPFNLDPEEGSASVRRWRDGSLEMDLPMIQLKELFNVNHFIVSQANPHIAPFLRMKEFVRACGGRFAAKLAQLAEMEVKHRCNQVLELGLPLREVASLFAQEWEGDVTIVMPATFSQYLKIIQNPSNVEIQKAANQGRRCTWEKLAVIKANFGIELALDECVTVLNHMRRLKRSAERAAAFSAISSSPPSKHLLAGTNRFNASKRIPSWNCIARQNSSGSVDDDVLAEASRLYQHIVVGSGRNSNRTSNLSHTYDAGSECDSPEAEDWTRSGGPLMRTNSAQMFTDYVQNLDAVDPEQIRASENDSIVAASSSSHSITVTEGDYLQTGRTHNGFVLNLVRGENLRMNSEPEDSQNESEIPETPESVQLDSPEKDIIDGESSASEDGDAQANLIHDHE

>ath:AT5G04040 SDP1;

MDISNEASVDPFSIGPSSIMGRTIAFRVLFCRSMSQLRRDLFRFLLHWFLRFKLTVSPFVSWFHPRNPQGILAVVTIIAFVLKRYTNVKIKAEMAYRRKFWRNMMRTALTYEEWAHAAKMLEKETPKMNESDLYDEELVKNKLQELRHRRQEGSLRDIMFCMRADLVRNLGNMCNSELHKGRLQVPRHIKEYIDEVSTQLRMVCNSDSEELSLEEKLSFMHETRHAFGRTALLLSGGASLGAFHVGVVRTLVEHKLLPRIIAGSSVGSIICAVVASRSWPELQSFFENSLHSLQFFDQLGGVFSIVKRVMTQGALHDIRQLQCMLRNLTSNLTFQEAYDMTGRILGITVCSPRKHEPPRCLNYLTSPHVVIWSAVTASCAFPGLFEAQELMAKDRSGEIVPYHPPFNLDPEVGTKSSSGRRWRDGSLEVDLPMMQLKELFNVNHFIVSQANPHIAPLLRLKDLVRAYGGRFAAKLAHLVEMEVKHRCNQVLELGFPLGGLAKLFAQEWEGDVTVVMPATLAQYSKIIQNPTHVELQKAANQGRRCTWEKLSAIKSNCGIELALDDSVAILNHMRRLKKSAERAATATSSSHHGLASTTRFNASRRIPSWNVLARENSTGSLDDLVTDNNLHASSGRNLSDSETESVELSSWTRTGGPLMRTASANKFIDFVQSLDIDIALVRGFSSSPNSPAVPPGGSFTPSPRSIAAHSDIESNSNSNNLGTSTSSITVTEGDLLQPERTSNGFVLNVVKRENLGMPSIGNQNTELPESVQLDIPEKEMDCSSVSEHEEDDNDNEEEHNGSSLVTVSSEDSGLQEPVSGSVIDA

>TcLPAT9 (XP_007028574)

MEISNEARVDSFLIGPSTIIGRTIAFRILFCKSLSHMRHQIFHVLLHFIYRCKDFLSPLVSWLHPRNPQGILAMVTIIAFLLKRYTNVKLRAEMAYRRKFWRNMMRTALTYEEWAHAAKMLDKETPKMNESDLYDEELVRNKLQELRHRRQDGSLRDIIFCMRADLIRNLGNMCNPELHKGRLHVPKLIKEYIDEVSTQLRMVCDSDSEELSLEEKLSFMHETRHAFGRTALLLSGGASLGAFHIGVVKTLVEHKLLPRIIAGSSVGSIMCSVVATRSWPELQSFFEDSWSSFQFFDQLGGIFSVVRRVMRQGAVHEIRQLQWMLRQLTSNLTFQEAYDMTGRILGITVCSPRKHEPPRCLNYLTSPHVVIWSAVTASCAFPVLFEAQELMAKDRSGEIVPYHPPFNLDPEEGSGISARRWRDGSLEVDLPMMQLKELFNVNHFIVSQANPHIAPLLRLKEFVRAFGGDFAAKLAQLTELEVKHRCHQILELGFPLGGLAKLFAQDWEGDVTVVMPATLAQYSKIIQNPSHLELQKAANQGRRCTWEKLSAIKANCGIELALDECVAILNHMRRLKRSADRAAASSHGLASTVRFNASKRIPSWNCIARENSTGSLEEDLTDVNSSLHQGVGGCTGIPPSGRNLRAHRSTHDGSDSESESVDVNSWTRSGGPLMRTTSANLFIDFVQNLDVDAEVNKGLMAHPSSPGFQMGGRDLLSHSSRVTTPDRGSEYEFDQRDLGNRTPVNGSSIMVTEGDLLQPERILNGFVLNVVKKEDLTLPHRILGSENYSAGVAECVQLDCPEKEMDASSASEYGDDATSEVNCLNETVPIVNATDDFSVHDDDRGVVDG

**DGAT genes used for phylogenetic tree construction of Fig 3c.**

>hsa:346606

MGVATTLQPPTTSKTLQKQHLEAVGAYQYVLTFLFMGPFFSLLVFVLLFTSLWPFSVFYLVWLYVDWDTPNQGGRRSEWIRNRAIWRQLRDYYPVKLVKTAELPPDRNYVLGAHPHGIMCTGFLCNFSTESNGFSQLFPGLRPWLAVLAGLFYLPVYRDYIMSFGLCPVSRQSLDFILSQPQLGQAVVIMVGGAHEALYSVPGEHCLTLQKRKGFVRLALRHGGPPHPRPPAPPPHRGGSQSLSRPLHDGPGAALRGAQGKLWGPRFHLPHLHLGLAAAFR

>TcDGAT2 (XP_007046425.1)

MMGEEMEERKATGYREFSGRHEFPSNTMHALLAMGIWLGAIHFNALLLLFSFLFLPFSKFLVVFGLLLLFMILPIDPYSKFGRRLSRYICKHACSYFPITLHVEDIHAFHPDRAYVFGFEPHSVLPIGVVALADLTGFMPLPKIKVLASSAVFYTPFLRHIWTWLGLTPATKKNFSSLLDAGYSCILVPGGVQETFHMEPGSEIAFLRARRGFVRIAMEMGSPLVPVFCFGQSHVYKWWKPGGKFYLQFSRAIKFTPIFFWGIFGSPLPYQHPMHVVVGKPIDVKKNPQPIVEEVIEVHDRFVEALQDLFERHKAQVGFADLPLKIL

>hsa:84649 DGAT2

MKTLIAAYSGVLRGERQAEADRSQRSHGGPALSREGSGRWGVACSAILMYIFCTDCWLIAVLYFTWLVFDWNTPKKGGRRSQWVRNWAVWRYFRDYFPIQLVKTHNLLTTRNYIFGYHPHGIMGLGAFCNFSTEATEVSKKFPGIRPYLATLAGNFRMPVLREYLMSGGICPVSRDTIDYLLSKNGSGNAIIIVVGGAAESLSSMPGKNAVTLRNRKGFVKLALRHGADLVPIYSFGENEVYKQVIFEEGSWGRWVQKKFQKYIGFAPCIFHGRGLFSSDTWGLVPYSKPITTVVGEPITIPKLEHPTQQDIDLYHTMYMEALVKLFDKHKTKFGLPETEVLEVN

>DGA1 (NP_014888.1)

MSGTFNDIRRRKKEEGSPTAGITERHENKSLSSIDKREQTLKPQLESCCPLATPFERRLQTLAVAWHTSSFVLFSIFTLFAISTPALWVLAIPYMIYFFFDRSPATGEVVNRYSLRFRSLPIWKWYCDYFPISLIKTVNLKPTFTLSKNKRVNEKNYKIRLWPTKYSINLKSNSTIDYRNQECTGPTYLFGYHPHGIGALGAFGAFATEGCNYSKIFPGIPISLMTLVTQFHIPLYRDYLLALGISSVSRKNALRTLSKNQSICIVVGGARESLLSSTNGTQLILNKRKGFIKLAIQTGNINLVPVFAFGEVDCYNVLSTKKDSVLGKMQLWFKENFGFTIPIFYARGLFNYDFGLLPFRAPINVVVGRPIYVEKKITNPPDDVVNHFHDLYIAELKRLYYENREKYGVPDAELKIVG

>TcDGAT3 (XP_007043617.1)

MGSEAQYKNAMALNGEEGEYSEPVSPTGQYFNSSALSIGVLGVLDSEIPIDDSPTMKLLEDVFLPINPRFSSVMVQDENGAKQWKKVEVKLVDHVNVPVFPPGLSPESYDNYLSDYLSKIATEQLPQNRPLWNIHIVKYTTSNAAGSLIFKLHHALGDGYSLMGALLSCLQRAENPSVPLTFPALSSAPNLSENSNSVFRSLYSAFNTISDFGWSLLKSSFLEDDRTMIRSGDPGVQFKPVVISTMTFSLDHIKQIKTKLGVTINDAITGIIFFGTRLYMQGTSSDKLSNGNSTALVLLNTRTIGGYKSVKDMVKPDADSPWGNQFGFLHVSLPELASTAESCNPLEFIWKAQKLIQRKRNSKAVFFTGQLLEGLRKYRGPEATAKYIHSTLKNSSMTISNLIGPVERMALADHPVKSLYFMVVGVPQSLTITMVSYMGKLTVAIGTEKDFVDPQKFKSSVENAFQMMLKAAQAIA

>AtDGAT:AT5G37300 WSD1

MKAEKVMEREIETTPIEPLSPMSHMLSSPNFFIVITFGFKTRCNRSAFVDGINNTLINAPRFSSKMEINYKKKGEPVWIPVKLRVDDHIIVPDLEYSNIQNPDQFVEDYTSNIANIPMDMSKPLWEFHLLNMKTSKAESLAIVKIHHSIGDGMSLMSLLLACSRKISDPDALVSNTTATKKPADSMAWWLFVGFWFMIRVTFTTIVEFSKLMLTVCFLEDTKNPLMGNPSDGFQSWKVVHRIISFEDVKLIKDTMNMKVNDVLLGMTQAGLSRYLSSKYDGSTAEKKKILEKLRVRGAVAINLRPATKIEDLADMMAKGSKCRWGNFIGTVIFPLWVKSEKDPLEYIRRAKATMDRKKISLEAFFFYGIIKFTLKFFGGKAVEAFGKRIFGHTSLAFSNVKGPDEEISFFHHPISYIAGSALVGAQALNIHFISYVDKIVINLAVDTTTIQDPNRLCDDMVEALEIIKSATQGEIFHKTEV

>TcDGAT4 (XP_007042465.1)

MACPGTFSDEPLTPAGRLFLQSQTNIIIHCILRGKNPIDIDAVKSTLRSSLMVRHPRFCSLLVRDKNGFEHWRKAQVDIDQHVIVIDKRLDKSDNFLDESRRFSSEEGEGDDDDEAAVNQYVADLSVSSPLSTDKPLWEIHILVPHKCAVFRIHHALGDGISLMSMLMASCRRADDADALPRMVPEKSAEFKDGKGRDWFWLFGILWGFLKMVCFTVMFVMEFVVSSLLVCDRKTVISGGEGVELWPRKLATARFLLEDMRVVKKAIPNTTINDVLFGVVSSGLSRYLDHRTPNALHEGLRMTGVAMVNLRPQTGFQDLSQLMKGAAEARWGNKFGLILLPVYFKKAGNNPLEYVKRAKRMVDRKKHSLEAYFSYRIGDLVMSLLGSKYACLLNYRLLCNTTFTISNIIGPLEEITLAGNPISSIKVNTSSLPQAITMHMLSYAGRAEMQILVAKDIVPDPEFLAKCFEDALLEMKEAVIGTEKE

>hsa:8694 DGAT1

MGDRGSSRRRRTGSRPSSHGGGGPAAAEEEVRDAAAGPDVGAAGDAPAPAPNKDGDAGVGSGHWELRCHRLQDSLFSSDSGFSNYRGILNWCVVMLILSNARLFLENLIKYGILVDPIQVVSLFLKDPYSWPAPCLVIAANVFAVAAFQVEKRLAVGALTEQAGLLLHVANLATILCFPAAVVLLVESITPVGSLLALMAHTILFLKLFSYRDVNSWCRRARAKAASAGKKASSAAAPHTVSYPDNLTYRDLYYFLFAPTLCYELNFPRSPRIRKRFLLRRILEMLFFTQLQVGLIQQWMVPTIQNSMKPFKDMDYSRIIERLLKLAVPNHLIWLIFFYWLFHSCLNAVAELMQFGDREFYRDWWNSESVTYFWQNWNIPVHKWCIRHFYKPMLRRGSSKWMARTGVFLASAFFHEYLVSVPLRMFRLWAFTGMMAQIPLAWFVGRFFQGNYGNAAVWLSLIIGQPIAVLMYVHDYYVLNYEAPAAEA

>TcDGAT1 (XP_007012778.1)

MAISDSPEILGSTATVTSSSHSDSDLNLLSIRRRTSTTAAGRAPDRDDSGNGEAVDDRDQVESANLMSNVAENANEMPNSSDTRFTYRPRVPAHRRIKESPLSSGAIFKQSHAGLFNLCIVVLVAVNSRLIIENLMKYGWLIRSGFWFSSRSLSDWPLFMCCLTLPIFPLAAFVVEKLVQRNYISEPVVVFLHAIISTTAVLYPVIVNLRCDSAFLSGVALMLFACIVWLKLVSYAHTNNDMRALAKSAEKGDVDPSYDVSFKSLAYFMVAPTLCYQQSYPRTPAVRKSWVVRQFIKLIVFTGLMGFIIEQYINPIVQNSQHPLKGNLLYAIERVLKLSVPNLYVWLCMFYCFFHLWLNILAELLRFGDREFYKDWWNAKTVEEYWRMWNMPVHKWMVRHIYFPCLRNGIPKGVAIVIAFLVSAVFHELCIAVPCHIFKLWAFIGIMFQVPLVLITNYLQDKFRSSMVGNMIFWFIFSILGQPMCVLLYYHDLMNRKGKAD

>TcDGAT6 (XP_007051549.1)

MESRNGFRWRKQGLKPIETKTGSKGEEENKSSSGQRTAEEEPLSPSARLFHEPNFNVYIIATLGCKTRIYPDVVKANLGHTLLKHPRFSSLQVEDEDNKGEMKWVRTEVDLERHVVVPDLDPNMDLPDKFLEDYIYNLSKTTIDKSQPLWDLHLLNLRTSQSEAVGVFRIHHSLGDGTSLMSLLLACTRQMNDPLALPTIPIRKKQERKNDRRGFWRIMFMLCSVFQVFWNSVVDVFMFMATALLLKDTENPFKGLPGVEFTPRRIVYGTVSLDDIKLVKNAMNTTVNDVALGITQAGLSRYINRIYGGNKNDGAAIEMDNLPKSIRLRSTLLVNIRPSAGIQALADMMEKDAEAKWGNWIGYVLLPFTIAIRDDPLDYVRDAKATIDRKKRSLEAIYTFSIAELVLKLFGVKAASALSHRIMSHTTMCFSNLVGPLEEIGFYGHPIAFLAPSSYGQPHALMINFQSYIDKMTIVLSVDEGTIPNPHQLCDDIVESLGLIKDAVVTRGLV

>TcDGAT8 (XP_007019909.1)

MDIIDLRSGSHLGLKQIKVTREMEEGRSVSGEDNEPLSPMARMFHEPDSNVYIITIVGFKNPIEPNSFKANLVHTLLKHPRFSSVQVADENNGGELKWVQTEVELEKHVIVPKVDEEMASQGAADKFIEDYISNMSKTKISMSIPMWDCHILNLKTSDAESVLVLRVHHSLGDGTSLMSLLISCSRKLFDPLALPTFPAMKKKPIATTTWLCFWIKLWSFFLLIWNTLVDMLMCVATLYFYKDTPTPLKPPSRSVACTPKRIVRRTFSLDDVKLVKNATNTLIWLIRVYLKCFAIGPFVDRQRCRPSNHSSRLISLDISTVNMRHEAGKEWEDNLPNNIRLRATLFINLRSSPGIYALGEMLKKNSKAEWGNKIGYVLFPFTIALKDNPLEYIRDVKEAMDRKKASLEAKFRHLMATVFVRFYRTRLAKFPSTTMWFSNVAGPQDEITIFGNQVTYIAPSLYGQPVALTIHVVSYAKKMSMVLSVDDNIIPDPYQLCDDLEESLKLIKKSVISQ

>AtDGAT:AT2G19450

MAILDSAGVTTVTENGGGEFVDLDRLRRRKSRSDSSNGLLLSGSDNNSPSDDVGAPADVRDRIDSVVNDDAQGTANLAGDNNGGGDNNGGGRGGGEGRGNADATFTYRPSVPAHRRARESPLSSDAIFKQSHAGLFNLCVVVLIAVNSRLIIENLMKYGWLIRTDFWFSSRSLRDWPLFMCCISLSIFPLAAFTVEKLVLQKYISEPVVIFLHIIITMTEVLYPVYVTLRCDSAFLSGVTLMLLTCIVWLKLVSYAHTSYDIRSLANAADKANPEVSYYVSLKSLAYFMVAPTLCYQPSYPRSACIRKGWVARQFAKLVIFTGFMGFIIEQYINPIVRNSKHPLKGDLLYAIERVLKLSVPNLYVWLCMFYCFFHLWLNILAELLCFGDREFYKDWWNAKSVGDYWRMWNMPVHKWMVRHIYFPCLRSKIPKTLAIIIAFLVSAVFHELCIAVPCRLFKLWAFLGIMFQVPLVFITNYLQERFGSTVGNMIFWFIFCIFGQPMCVLLYYHDLMNRKGSMS

>TcDGAT5 (XP_007039033.1)

MNRKSEAKWVPKLFHQSLISMNSTEIQEIDQVEAKIGTEEASPVSKDEVPLSTGSLIFHEPGINCCIIAIMGYKSKLDPAVIKEGLKQTLIKHPCFSSKLLSAIDQMMNSGKKKWIRTQVNVDNHVILPEVDTVTESPSQFIEDYVTYLTGIPIDLSKPLWELHLVNLKTPEAEAVGIFRIHHSIGDGMSLISLLLACCRKSSDPKALPTLPKQKQADSRYPHPHGFFRLFLAIWSVLRLILNTLVDLLLFVATVVFLKDTKTPLKGSSGVEQNAKKIVHRTVSLDDIKLVKDAMGMTVNDVILGVTEAGLSRYLNRKYGEVDRGKEAEQKSNHLPRNIRLRATALVNIRQTAGIQVSFLSPFSRASEKVEYASQSFLVFKSKVKWGNQIGYICIPFTIALRNDPLDYLRGAKAAGDRKKLSLEAICTHLTNKCVVKLFGSKLSAALVYRVIFNTTMTLSNVVGPVEEISLYGHPIAFIAPSVYGHPQALTCHFQSYMNKMSIVLAVDPNVIPDPRLLCDDLEESLKIFKDAVVLAKDAD

>TcDGAT7 (XP_007019907.1)

MDIIGLRSRSHLGLKQIKVTRDMKEGQSVSGEEEEPLSPMARMFHEPDSNVKEINDALHAIPMILQTLTCSNMNSAYRQGRRVADENNGGELKWVKTEVELEKHVIIPRVDEEMASQGAADKFVEDYIANISKTKISLSIPMWDCHILNIKTSDAESVLVLLVHHSLGDGTSLMSLLISCSRKLSDLLALLTFPAMKKKQMPTTTTTTWLCFWIRLWSFFLLIWNTSVDLLVCVATLFFYKDTPTPLKPSSRRVACTPGRIMRRTFTLDDIKLVKNATNMTVNDVVLAITQAGLSRYLNRKYGKTKRNEAGRKWEDNLPNNIRLRATLFINLRSSPGIYALGEMLKKNSKAEWGNKIGYVLFPFTISLKDNLLDYIRDVKAATDRKKAYLEAKFRLLMAMVFVRFYRTKFAKFPSTTMWFSNVARPQDDITIFGNQVAYIAPSLYGQLVALTVHVVSYAKMISMVLSVDDNIISDPYQLCDDLEESLKLIKTSVVSPLVRVRLSYSRTKSRSTLVILAKFPSTTIWFSNVARPQDEITIFGNQVAYIASSVYGQPKALTIHVVSFAKKISQPVVLLAGHPVPLTPMLPRRAGADASL

>LRO1 (NP_014405.1)

MGTLFRRNVQNQKSDSDENNKGGSVHNKRESRNHIHHQQGLGHKRRRGISGSAKRNERGKDFDRKRDGNGRKRWRDSRRLIFILGAFLGVLLPFSFGAYHVHNSDSDLFDNFVNFDSLKVYLDDWKDVLPQGISSFIDDIQAGNYSTSSLDDLSENFAVGKQLLRDYNIEAKHPVVMVPGVISTGIESWGVIGDDECDSSAHFRKRLWGSFYMLRTMVMDKVCWLKHVMLDPETGLDPPNFTLRAAQGFESTDYFIAGYWIWNKVFQNLGVIGYEPNKMTSAAYDWRLAYLDLERRDRYFTKLKEQIELFHQLSGEKVCLIGHSMGSQIIFYFMKWVEAEGPLYGNGGRGWVNEHIDSFINAAGTLLGAPKAVPALISGEMKDTIQLNTLAMYGLEKFFSRIERVKMLQTWGGIPSMLPKGEEVIWGDMKSSSEDALNNNTDTYGNFIRFERNTSDAFNKNLTMKDAINMTLSISPEWLQRRVHEQYSFGYSKNEEELRKNELHHKHWSNPMEVPLPEAPHMKIYCIYGVNNPTERAYVYKEEDDSSALNLTIDYESKQPVFLTEGDGTVPLVAHSMCHKWAQGASPYNPAGINVTIVEMKHQPDRFDIRGGAKSAEHVDILGSAELNDYILKIASGNGDLVEPRQLSNLSQWVSQMPFPM

>ath:AT3G44830

MSPLLRFRKLSSFSEDTINPKPKQSATVEKPKRRRSGRCSCVDSCCWLIGYLCTAWWLLLFLYHSVPVPAMLQAPESPGTRLSRDGVKAFHPVILVPGIVTGGLELWEGRPCAEGLFRKRLWGASFSEILRRPLCWLEHLSLDSETGLDPSGIRVRAVPGLVAADYFAPCYFAWAVLIENLAKIGYEGKNLHMASYDWRLSFHNTEVRDQSLSRLKSKIELMYATNGFKKVVVVPHSMGAIYFLHFLKWVETPLPDGGGGGGPGWCAKHIKSVVNIGPAFLGVPKAVSNLLSAEGKDIAYARSLAPGLLDSELLKLQTLEHLMRMSHSWDSIVSLLPKGGEAIWGDLDSHAEEGLNCIYSKRKSSQLSLSNLHKQNYSLKPVSRVKEPAKYGRIVSFGKRASELPSSQLSTLNVKELSRVDGNSNDSTSCGEFWSEYNEMSRESIVKVAENTAYTATTVLDLLRFIAPKMMRRAEAHFSHGIADDLDDPKYGHYKYWSNPLETKLPEAPEMEMYCLYGVGIPTERSYIYKLATSSGKCKSSIPFRIDGSLDGDDVCLKGGTRFADGDESVPVISAGFMCAKGWRGKTRFNPSGMDTFLREYKHKPPGSLLESRGTESGAHVDIMGNVGLIEDVLRIAAGASGQEIGGDRIYSDVMRMSERISIKL

>TcDGAT9 (XP_007012701.1)

MSFLRRRKVTDSSKSQNSDSNAGKEDDKKKQYKSPKRESYSSKKWSCWDSCCWFIGLICSMWWFLLFLYNAMPASIPQYVTEAITGPLPDPPGVKLRKEGLTVNHPVVFVPGIVTGGLELWEGHHCADGLFRKRLWGGSFGELYKRPLCWAEHMSLDNETGLDPPGIRVRPVSGLVAADYFAAGYFVWAVLIANLAQIGYEEKTMYMAAYDWRLSFQNTEVRDQTLSRIKSNIELMVATNGGKKVVVIPHSMGVLYFLHFMKWVEAPVPMGGGGGSDWCAKHIKAVMNIGAPFLGVPKSVSGLFSIEARDIAIARAFAPGFLDKDVFGLQTFQHLMRMTRTWDSTMSMIPKGGDTIWGGLDWSPEGGSFNCSAKKLKNNGTRTGHNANSNLGNMKSVNYGRIISFGKDVAEAHSSKIERVDFRDVMKGGKLANSSNCDIWTEYHEMGNGAIKAVADYKVYTAGSILDLLHFVAPKLMARGGAHFSYGIADNLDDPKYEHYKYWSNPLETKLPSAPDMEIYSMYGVGIPTERAYVYKLTTATDCYIPFQIDTSAEGGSEGSCLKGGVFSVDGDETVPVLSAGFMCAKGWRGKTRFNPSGIRTYIREYNHAPPSNLLEGRGTQSGAHVDIMGNFALIEDIIRVAAGATGQDLGGDRVYSDIFKWSERINLQL

>ath:AT5G13640

MPLIHRKKPTEKPSTPPSEEVVHDEDSQKKPHESSKSHHKKSNGGGKWSCIDSCCWFIGCVCVTWWFLLFLYNAMPASFPQYVTERITGPLPDPPGVKLKKEGLKAKHPVVFIPGIVTGGLELWEGKQCADGLFRKRLWGGTFGEVYKRPLCWVEHMSLDNETGLDPAGIRVRAVSGLVAADYFAPGYFVWAVLIANLAHIGYEEKNMYMAAYDWRLSFQNTEVRDQTLSRMKSNIELMVSTNGGKKAVIVPHSMGVLYFLHFMKWVEAPAPLGGGGGPDWCAKYIKAVMNIGGPFLGVPKAVAGLFSAEAKDVAVARAIAPGFLDTDIFRLQTLQHVMRMTRTWDSTMSMLPKGGDTIWGGLDWSPEKGHTCCGKKQKNNETCGEAGENGVSKKSPVNYGRMISFGKEVAEAAPSEINNIDFRGAVKGQSIPNHTCRDVWTEYHDMGIAGIKAIAEYKVYTAGEAIDLLHYVAPKMMARGAAHFSYGIADDLDDTKYQDPKYWSNPLETKLPNAPEMEIYSLYGVGIPTERAYVYKLNQSPDSCIPFQIFTSAHEEDEDSCLKAGVYNVDGDETVPVLSAGYMCAKAWRGKTRFNPSGIKTYIREYNHSPPANLLEGRGTQSGAHVDIMGNFALIEDIMRVAAGGNGSDIGHDQVHSGIFEWSERIDLKL

>TcDGAT11 (XP_007024579.1)

MSAIRRRKPINESDKTSEASDSKPHKEKEEEQHGDDDGSDKDKKKIPSKIKKKHEEKQPKWSCLDSCCWFIGCICVTWWLLLFLYNAMPASFPQYVTEAITGPLPDPPGVKLKKEGLKAKHPVVFVPGIVTGGLELWEGRKCAEGLFRKRFWGGTFGEVYKRPLCWVEHMSLDNETGLDPCGIRVRPVSGLVAADYFAPGYFVWAVLIANLARIGYEEKTMYMAAYDWRLSFQNTEARDQTLSRIKSNIELIVATNGGKKAVVIPHSMGVLYFLHFMKWVEAPAPMGGGGGPDWCSKHIKAVVNIGGPFLGVPKAIAGLFSAEAKDIAVVRAIAPGFLDNDIFQLQTLQHVMRMSRSWDSTMSMIPRGGDTIWGGLDWSPEEGYSCAKKRERKNDTQIAEEAGVESAVSQTRSAKYGRIISFGKDVAEAPSSDVERIDFRDAVKGHSAANTTCRDVWTEYHDMGFGGINAVAEYKTYTAESIVDLLHFVAPKMMARGTAHFSYGIADNLDDPKYKHYKYWSNPLETKLPNAPEMEIFSLYGVGLPTERAYIYKLSPAAECYIPFQIDTSADDEETCLKDGVYSVDGDETVPVLSAGFMCAKGWRGKTRFNPSGIRTYIREYSHSPPANLLEGRGTLSGAHVDIMGNFALIEDVIRVAAGASGEELGGDQVYSNIFNWSEKIDLQL

>TcDGAT10 (XP_007015990.1)

MASILRFRKLCYVEQAVKCASVGFESFDQSPKIDQKLDKKEEEVISANNFALEIIKKRKQPRKQPKEWRRLDSCCWMIGYLCSTWWLLLFLYHSLPVTLLQVPESPGVRLKREGLAALHPVVLVPGIVTGGLELWEGQPCADGLFRKRLWGGSFTEIFKRPLCWLEHLSLHSETGLDPPGIRVRAVPGLVAADYFAPGYFVWAVLIENLAKIGYEGKNLHMAAYDWRLSFQNTEIRDHALSRLKSKIELMYLTNGYKKVAVVPHSMGVIYFLHFLKWVETPPPIGGGGGPGWCAKHIKAIMNIGPAFLGVPKAVSNIFSAEGKDIAYIRAMAPGLLDSKILGLQTLEHVMRVSRTWDSIVSLVPKGGETIWGNMDWSPEERHACDFSRKRHSQPSPIDNNVNNSDVKRGFRVKDPVQYGRIISFGKTASQLHSSQLPTIDSKEFLRTSASQNLNFSCGEAWTEYDEMSREGIQKVAANKAYTTQTLLDLLRFVAPKMMLRAEAHFSHGIADDLDNPKYNHYKYWSNPLEMKLPDAPDMEIYCLYGVGIPTERSYVYKLSPTSGCKSIPYQIDSSVNGEDGSCLKGGVYFADGDENVPVLSAGFMCAKGWRGRTRFNPSGIATYIREYRHKPPSSLLEGRGIESGAHVDIMGNVALIEDILRIASGATGKEIRGDRIYSDILRMSERINLRL
